# Oligosaccharides from *Polygonatum cyrtonema* Hua ameliorate colitis-induced lung injury via modulation of the gut-lung axis through NF-κB and Nrf2 pathways

**DOI:** 10.1007/s13659-026-00608-0

**Published:** 2026-06-23

**Authors:** Jin Xu, Chuankang Tang, Qianyu He, Yu Lu, Pei Luo, Jianbo Wu

**Affiliations:** 1 Department of Science and Technology, Department of Gastroenterology, Luzhou People’s Hospital, Luzhou, 646000 China; 2 Department of Gastroenterology, Affiliated Hospital of Southwest Medical University, Luzhou, 646000 China; 3 Basic Medicine Research Innovation Center for Cardiometabolic Diseases, Ministry of Education, Laboratory for Cardiovascular Pharmacology, Department of Pharmacology, School of Pharmacy, Luzhou Municipal Key Laboratory of Thrombosis and Vascular Biology, Southwest Medical University, Luzhou, 646000 China; 4 Faculty of Chinese Medicine, Macau University of Science and Technology, Avenida Wai Long, Macau, People’s Republic of China; 5 State Key Laboratory of Quality Research in Chinese Medicine (Macau University of Science and Technology), Avenida Wai Long, Macau, People’s Republic of China

**Keywords:** PFOS, Colitis-induced lung injury, NF-κB/IKK-β, Nrf2/HO-1/NQO-1, Mucosal barrier, Intestinal metabolites

## Abstract

**Background:**

The gut-lung axis is a bidirectional communication network linking intestinal and pulmonary homeostasis through shared immunological and molecular mechanisms, conceptually consistent with the traditional Chinese medicine theory of “lung-intestine combined treatment.” *Polygonatum cyrtonema* Hua is an edible medicinal plant with reported anti-inflammatory and antioxidant properties, yet its role in intestinal inflammation-associated lung injury remains unclear.

**Materials and methods:**

Dextran sulfate sodium (DSS)-induced colitis and lipopolysaccharide (LPS)-induced lung injury models were established in vivo, while LPS-stimulated A549 lung epithelial cells were used in vitro. The protective effects of *Polygonatum cyrtonema* oligosaccharides (PFOS) were evaluated, with particular focus on NF-κB and Nrf2 signaling pathways. The Nrf2 inhibitor ML385 was applied in vitro to verify pathway involvement. Non-targeted fecal metabolomics was conducted to assess PFOS-mediated metabolic modulation.

**Results:**

PFOS significantly alleviated DSS-associated lung histopathological damage, reduced inflammatory cell infiltration, and improved epithelial barrier integrity. PFOS suppressed pulmonary proinflammatory cytokines, including TNF-α and IL-6, decreased myeloperoxidase activity, and attenuated oxidative stress by lowering malondialdehyde levels while enhancing antioxidant enzymes such as superoxide dismutase and HO-1. Mechanistically, PFOS inhibited NF-κB activation and promoted Nrf2/HO-1 signaling in lung tissues and LPS-stimulated A549 cells, effects that were partially reversed by ML385. Metabolomics analysis revealed that PFOS corrected DSS-induced disturbances in amino acid and lipid metabolism, with enrichment in cAMP, PPAR, and tryptophan-related pathways.

**Conclusions:**

PFOS protects against colitis-associated lung injury by modulating the gut-lung axis through coordinated anti-inflammatory and antioxidant mechanisms involving NF-κB inhibition and Nrf2 activation, supporting its potential therapeutic application in gut-lung axis-related diseases.

**Supplementary Information:**

The online version contains supplementary material available at 10.1007/s13659-026-00608-0.

## Highlights

1. PFOS may affect the formation of tight epithelial barrier junctions by promoting ZO-1 and Claudin-1 separation, thereby reducing lung injury associated with enteritis.
2. PFOS administration suppressed NF-kB signaling pathway and up regulated Nrf2 signaling pathway to reduce inflammation and oxidative stress.
3. PFOS inhibited LPS-induced epithelial barrier damages, inflammation and oxidative stress in lung epithelial cells A549.
4. Intestinal metabolites affected PFOS exhibits potential correlations to the lung inflammation.

**Supplementary Information:**

The online version contains supplementary material available at 10.1007/s13659-026-00608-0.

## Introduction

The gut-lung axis represents a bidirectional communication network linking the lungs and intestines through shared embryonic origins, a common mucosal immune system, and overlapping molecular and immunomodulatory mechanisms [1, 2]. Key processes, including inflammatory mediator circulation, gut microbiota dysbiosis, and oxidative stress, underpin this axis and contribute to the pathogenesis of diseases affecting both organ systems, such as infections and chronic inflammatory conditions [3, 4]. Clinical evidence further supports the gut-lung axis, with 39.6% of SARS-CoV-2 patients in Wuhan exhibiting gastrointestinal symptoms [5] and the RHINE study reporting a higher prevalence of asthma and respiratory symptoms in patients with inflammatory bowel disease (IBD), particularly those with ulcerative colitis(UC) [6]. The classic theory of traditional Chinese medicine "the lung stands in interior-exterior relationship with the large intestine" also elaborates the intimate connection between the intestines and lungs [7]. At present, the treatment plans for UC rarely manage to address both the extraintestinal symptoms and the respiratory symptoms of some UC patients. Obviously, this field is worthy of further exploration.

Polygonatum is widely valued for its medicinal and nutritional properties, serving as a traditional tonic and therapeutic agent in countries such as China, Pakistan, Iran, and Japan [8]. Its bioactive constituents, including steroidal saponins, flavonoids, polysaccharides, and alkaloids, have been extensively studied [9]. Previously, we successfully isolated and characterized oligosaccharides from *Polygonatum cyrtonema* Hua (PFOS) and demonstrated their anti-inflammatory and antioxidant effects in mitigating dextran sodium sulfate (DSS)-induced intestinal damage, lipopolysaccharide (LPS)-induced peritonitis, and acute lung injury in mice [10, 11]. Additionally, DSS-induced colitis in susceptible models has been shown to trigger lung inflammation and tissue damage [12]. Since we have demonstrated the protective effects of PFOS on the gut and lung, we were curious about the therapeutic potential of PFOS in DSS-induced enteritis-associated lung injury.

Nuclear factor-erythroid 2-related factor 2 (Nrf2) is a pivotal regulator of antioxidant defenses and modulates over 200 genes, including those encoding glutathione peroxidase (GPx), glutathione (GSH), and heme oxygenase-1 (HO-1) [13, 14]. Nrf2 activation also inhibits ferroptosis, alleviating intestinal ischemia/reperfusion-induced lung injury [13, 15], while cigarette smoke-induced reactive oxygen species (ROS) in chronic obstructive pulmonary disease (COPD) may exacerbate intestinal disorders [3]. Hence, we speculate that Nrf2 plays an important role in the diseases related to gut-lung crosstalk. Furthermore, emerging evidence highlights the anti-inflammatory role of the Nrf2/HO-1 pathway, with compounds such as sinomenine promoting M2 macrophage polarization and inhibiting NF-κB activation via Nrf2-dependent mechanisms [16, 17]. Taken together, to activate Nrf2 signaling pathway may contribute to anti-oxidant and anti-inffammatory for treating colitis-induced lung injury.

We established a DSS-induced mouse model of enteritis-associated lung injury, and employed a lung epithelial cell model to elucidate the effects of PFOS on lung pathology in the context of enteritis. Using advanced biotechnological approaches, we evaluated the efficacy and mechanisms of PFOS, focusing on its modulation of oxidative stress and inflammatory responses via the Nrf2 and NF-κB signaling pathways. Notably, the analysis of KEGG enrichment pathways revealed that PFOS may regulate the NF-κB and Nrf2 signaling pathways by enriching the metabolites of cAMP, PPAR, and TRP pathways.

## Materials and methods

### Materials

*Polygonatum cyrtonema* Hua was collected from Renshou country (sichuan province, China) and identified by Professor Zhifeng Zhang (Macau University of Science and Technology, China). The original plant specimen is kept at Faculty of Chinese Medicine, Macau University of Science and Technology, its number is must-01034. PFOS was prepared as previous report [10]. Dextran sulfate sodium (DSS) was obtained from Yuanye Biotechnology Co. Ltd. (S14049, molecular weight: 50,000, Shanghai, China). The CAT, SOD, MDA, and GPX2 assay kits were purchased from JSBOSSEN Biotechnology Company (Nanjing, China) or Jiancheng Biotechnology Company (Nanjing, China). Human IL-1β, IL-6, TNF-α, and IL-10 ELISA kits were purchased from JSBOSSEN. The primary antibodies against ZO-1,Claudin-1, p65,p-p65, IKK-β, Nrf2,HO-1,NQO-1, Keap-1were from Santa Cruz or Proteintech. The Nrf2 inhibitor, ML385, was purchased from MCE.

### Animals and treatment

Ethical statement. The animal procedures in this study followed the “Principles of Laboratory Animal Care” published by the National Society for Medical Research and the “Guide for the Care and Use of Laboratory Animals” developed by the Institute of Laboratory Animal Resources and published by the National Institutes of Health in 2011 (8th edition). All experimental procedures were conducted in strict accordance with guidelines approved by the Animal Ethics Committee of Southwest Medical University (20240229-01). All reported methods were in accordance with the ARRIVE guidelines.

Male C57BL/6 mice (6–8 weeks old) obtained from Chengdu Yaokang Biotechnology Co., Ltd. were used for all studies. All mice were maintained in a room with a 12 h/12 h light/dark cycle at a constant temperature (20–25 ℃) and allowed access to water and food ad libitum. After an acclimatization period of 1 week, the mice were grouped according to their weight. Mice were divided into four groups: Group 1 (normal control), no DSS exposure and no treatment; Group 2 (negative control), DSS exposure with the same volume of PBS treatment; and Groups 3–4: DSS exposure and 0.5 and 2 mg/Kg/d PFOS treatment. Mice were orally gavaged with the same volume of PBS or PFOS for 2 weeks. Acute colitis was induced by administering 3% DSS salt to mice for 7 days. Briefly, DSS was dissolved in drinking water to a concentration of 3% and the mice were allowed to drink freely. The DSS solution was replaced daily. Eighth day, the mice were euthanized with 1% sodium pentobarbital administered via intraperitoneal injection.

### Histological analysis

Lung tissues were fixed in 4% neutral formaldehyde for 24 h, embedded in paraffin, and cut into 2 µm sections. Sections dewaxed in water were stained with hematoxylin and eosin (H&E), followed by microscopic observation. The Smith score was used to evaluate lung injury [18]. Briefly, the pathological injury score of lung tissue was used to evaluate the degree of hemorrhage and edema in six different fields of the same section: no injury, 0 score; damage area less than 25%, 1 score; the damage area was 25–50%, 2 points; the damage area was 50–75%, 3 points; and more than 75% of the damage. Four points. The average total score for each group was divided into the lung injury scores.

### Immunohistochemical analysis

After the lung sections were repaired in citrate buffer at high temperature, H_2_O_2_ was added to block the removal of endogenous enzymes. After the primary antibody (4) was added overnight, the secondary antibody was added and incubated for 45 min. Diluent cell signaling11724S with DAB(kit, sigalstainR DAB Diluent cell signaling11724S) and hematoxylin staining, and images were obtained using a light microscope (Olympus, Japan).

### Immunofluorescence

The lung sections were fixed with paraformaldehyde for 10 min, EDTA was used for antigen repair, BSA was added, and the serum was blocked for 30 min, followed by incubation at 4 °C overnight and incubation at room temperature for 50 min. The DAPI dye solution was added to the lung sections and incubated at room temperature for 10 min in the dark. The slices were slightly dried and sealed with an anti-fluorescence attenuation sealant. Images were obtained using a fluorescence microscope. According to the results of the immunofluorescence experiments, nuclei stained with DAPI were blue under ultraviolet excitation, and the positive expression was luciferin-labeled green.

### Cell culture and cell viability assay

The human alveolar epithelial cell line A549 (purchased from Guangzhou Rongman Biotechnology Co., LTD.) requires a complete culture medium consisting of DMEM high-glucose medium, 1% penicillin–streptomycin double-antibiotic solution, and 10%FBS, cultured in a 5%CO_2_ incubator at 37 °C. The optimal concentration of PFOS in LPS-induced A549 pneumonia was determined using the CCK-8 method. Briefly, logarithmic cells were collected and 96-well plates were inoculated at a cell density of 1,000–10,000 cells/well. After 24 h of incubation, seven PFOS concentrations (0.3125 μM, 0.625 μM, 1.5 μM, 5 μM, 10 μM, 15 μM and 20 μM), respectively. Additionally, 1 μg/mL LPS was administered for 24 h. After 24 h, take out the 96-well plate and add 10μL CCK-8 solution was added to each well. Incubation was continued in the cell incubator, and the OD value of each well at 450 nm was measured using an enzyme enzyme-labeler after 1 h and 2 h of incubation, respectively. Based on the OD value, the optimal concentration of oligosaccharides was selected to continue the sequential experiments.

### Cell experimental

A549 cells (2 × 10^5^ cells/ml) were seeded in 6-well plates, and then cultured at 37.0℃ with 5% CO_2_ for 24 h. Cells were treated with PFOS (5 μM) for 12 h, subsequently with 1 μg/mL LPS for 24 h with or without ML385 (10 μM, a specific inhibitor of Nrf2) for 2 h before. Cell suspensions were collected and stored at − 80 ℃ to detect antioxidant activity. The cell samples were collected for protein analysis.

### Western blot analysis

RIPA pyrolysis liquid containing 10ul PMSA protease inhibitor was added to 100 mg of lung tissue or cell extract and ground on ice until the tissue was completely lysed. The lysed sample was centrifuged at 12,000 rpm for 20 min at 4 °C and the supernatant was collected. Add 25ul loading buffer (SDS) was added to the supernatant, and the 816 metal bath was heated at 95 °C and boiled for 8 min. Equal amounts of protein (45 μg) were loaded onto 10% sodium dodecyl sulfate–polyacrylamide gel electrophoresis (SDS-PAGE) and transferred to polyvinylidene difluoride (PVDF) membranes. The membrane was blocked with a 5% skim milk powder solution for 1 h and then incubated overnight at 4 ℃with specific primary antibodies. Horseradish peroxidase (HRP)-coupled secondary antibodies were then added and incubated for 1.5 h at room temperature. Bands were visualized by chemiluminescence using an Omni-ECLFemto Light Chemiluminescence Kit. Protein expression was quantified using the ImageJ software.

### Enzyme-linked immunosorbent assay

The levels of inflammatory cytokines in the supernatants of A549 cells treated with LPS and the levels of oxidative stress-related indicators in the cell extract were quantified using human ELISA kits (JSBOSSEN, Nanjing, China) according to the manufacturer’s instructions. MPO levels in lung tissue homogenates were quantified using mouse ELISA kits (Jiancheng, Nanjing, China).

### Detection of oxidative stress related indicators activity in lung tissue

The activity levels of SOD, GPX2, CAT, and MDA in the lung tissues were detected using biochemical detection kits, according to the manufacturer’s instructions.

### Quantitative real-time PCR

Total RNA was extracted from the lung tissues using TRIZOL reagent (Invitrogen, USA). cDNA synthesis was performed using the PrimeScript^TM^ RT Reagent Kit (Takara, Japan) and gDNA Eraser (Perfect Real Time) Kit (Takara, Japan). Real-time PCR was performed using the SYBR ^®^ RT-qPCR kit (Takara, Japan) according to the manufacturer’s instructions. Reaction conditions: pre-denaturation (95 ℃, 2 min), denaturation (95 ℃, 5 s), annealing (55–60 ℃, 10 s), amplification (55 cycles), extension (55–95 ℃), and melting point curve analysis. All primers used in this study are listed in Supplementary Table S1.

### Non-targeted metabolomics analysis of cecal metabolites

Each specimen (50 mg) was weighed into a 2 mL centrifuge tube, and a 6 mm diameter grinding bead was added. A 400μL mixture of methanol and water was prepared in a 4:1 volume ratio, and 0.02 mg/mL of L-2-chlorophenylalanine was added to the mixture to prepare the extraction solution, which was then added to the centrifuge tube. The sample solution was ground in a frozen tissue grinder for 6 min (−10 ℃, 50 Hz) and then extracted by ultrasound at a low temperature for 30 min (5 ℃, 40 kHz). The sample was placed at −20 ℃ for 30 min, centrifuged for 15 min (4 ℃, 13,000 × g), and the supernatant was transferred to an injection vial with internal intubation for machine analysis. The samples were analyzed by LC–MS/MS using a Thermo Field ultra-high-performance liquid chromatography tandem Fourier transform mass spectrometry UHPLC-Q Exactive HF-X system. Chromatographic conditions were set: 3ul samples were separated using an HSS T3 column (100 mm × 2.1 mm i.d., 1.8 µm) and then subjected to mass spectrometry for detection. Mobile phase A consisted of 95% water + 5% acetonitrile (containing 0.1% formic acid) and mobile phase B consisted of 47.5% acetonitrile + 47.5% isopropyl alcohol + 5% water (containing 0.1% formic acid). The flow rate was 0.40 mL/min and the column temperature was 40 ℃. Mass spectrum conditions: The sample quality spectrum signal was collected in positive and negative ion scanning modes, and the quality scanning range was 70–1050 m/z. The flow rate of the sheath gas was 50 psi, the flow rate of the auxiliary gas was 13 psi, the heating temperature of the auxiliary gas was 425 ℃, the positive mode ion spray voltage was 3500 V, the negative mode ion spray voltage was set to −3500 V, the ion transport tube temperature was 325 ℃, and the normalized collision energy was 20-40-60 V. The primary mass spectrometry resolution was 60,000, the secondary mass spectrometry resolution was 7500, and the data were collected in DDA mode. After completion of the computer, the LC–MS raw data were imported into metabolomics processing software for analysis.

### Statistical analysis

All experiments were repeated three or more times, and the data for each group are expressed as mean ± SD or mean ± SEM. SPSS21.0 was used for paired t-test and one-way statistical analysis of variance. #p < 0.05, ##p < 0.01, ###p < 0.001, indicating that pairwise comparisons were statistically significant; ns p > 0.05, indicating no statistical significance.

## Results

### PFOS attenuates DSS-induced enteritis-associated lung injury

**Fig. 1:**
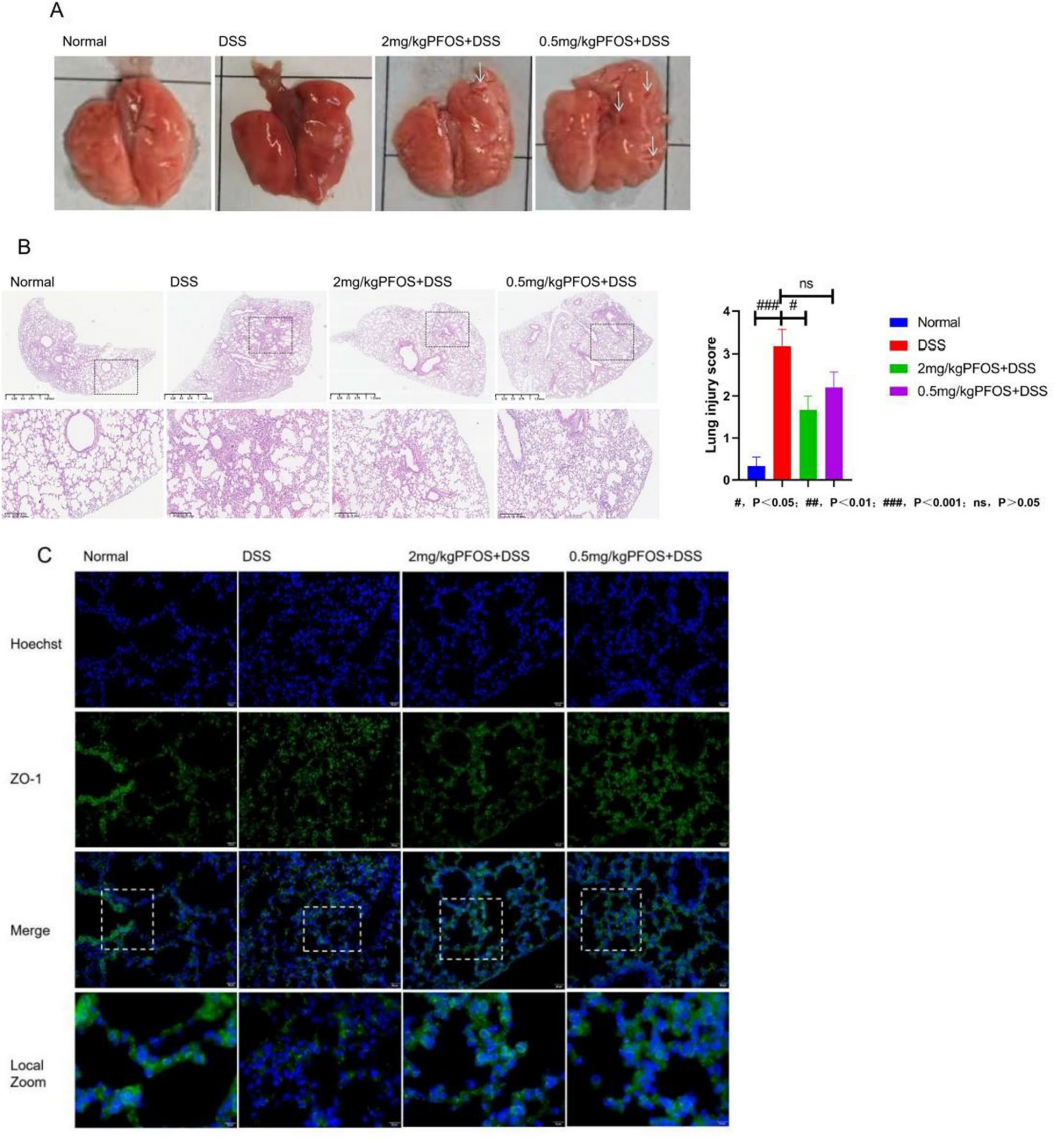

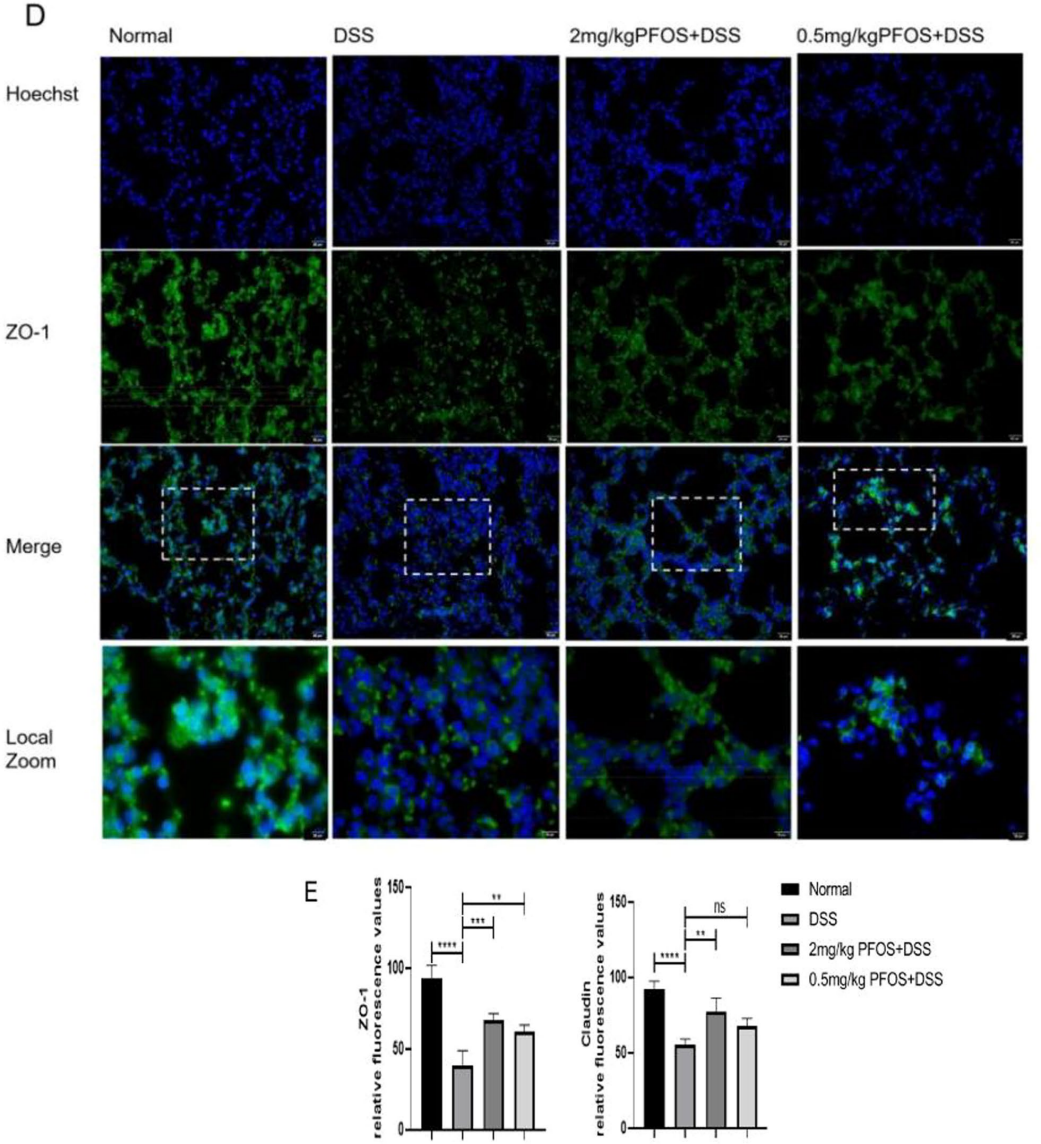
PFOS reduces lung damage associated with DSS-induced enteritis. **A** Representative images of the lung macrograph. **B** The most representative HE staining diagram (magnification 20 × and 100 ×) of each experimental group shows typical lung tissue injury changes, including alveolar structure rupture and fusion, a large number of inflammatory cells and red blood cell infiltration, pulmonary interstitial hyperemia and edema, and diffuse thickening. **C**, **D** Representative immunofluorescence staining of ZO-1 and Claudin-1 in colon sections as indicated(bar, 20 μm and 10 μm). n = 5–8/group. **E** The statistical results of the average fluorescence intensity of ZO-1 and Claudin-1. Data are shown as the mean ± SEM. #p < 0.05, ##p < 0.01, ###p < 0.001

Intestinal barrier dysfunction is a recognized contributor to acute lung injury (ALI) in extrapulmonary diseases [19]. Building on our previous findings of PFOS on the intestinal barrier [11], we evaluated its efficacy in mitigating lung injury in a dextran sodium sulfate (DSS)-induced enteritis mouse model. Gross examination of lung tissues revealed distinct differences between the groups (Fig. 1A). The lungs of the control group displayed a glossy and uniformly pink appearance with elasticity, whereas those of the DSS model group appeared dark red, dull, and swollen. Pre-administration of PFOS (two doses) in DSS-treated mice markedly improved lung appearance, showing a pink color with scattered hemorrhagic spots and retained glossiness. Hematoxylin and eosin (HE) staining confirmed reduced lung damage in PFOS-treated mice, with significantly lower histopathological scores reflecting decreased inflammatory cell infiltration compared to the DSS group (Fig. 1B). Immunofluorescence analysis of tight junction proteins ZO-1 and Claudin-1 showed that PFOS pre-administration reduced cytoplasmic droplet accumulation and partially restored the ring-like membrane structure (Fig. 1C–E). Given that ZO-1 phase separation drives tight junction formation [20], these findings suggest that PFOS enhances epithelial barrier integrity, thereby alleviating enteritis-associated lung injuries.

In our previous study [11], PFOS significantly ameliorated DSS-induced colitis, as evidenced by improved colon histopathology, reduced inflammatory cell infiltration, and decreased expression of pro-inflammatory cytokines, accompanied by restoration of antioxidant capacity and intestinal barrier integrity. In the current study, although detailed colon pathology was not re-evaluated, PFOS administration similarly resulted in marked attenuation of lung injury, including reduced inflammatory infiltration, decreased pulmonary cytokine expression, and improved oxidative stress parameters.

Taken together, these findings suggest that the protective effects of PFOS on lung tissue occur concomitantly with its previously established anti-inflammatory and antioxidant effects in the colon, supporting the concept that mitigation of intestinal inflammation is a critical upstream event in preventing colitis-associated lung injury.

### PFOS suppresses lung inflammation in DSS-induced colitis mice

**Fig. 2:**
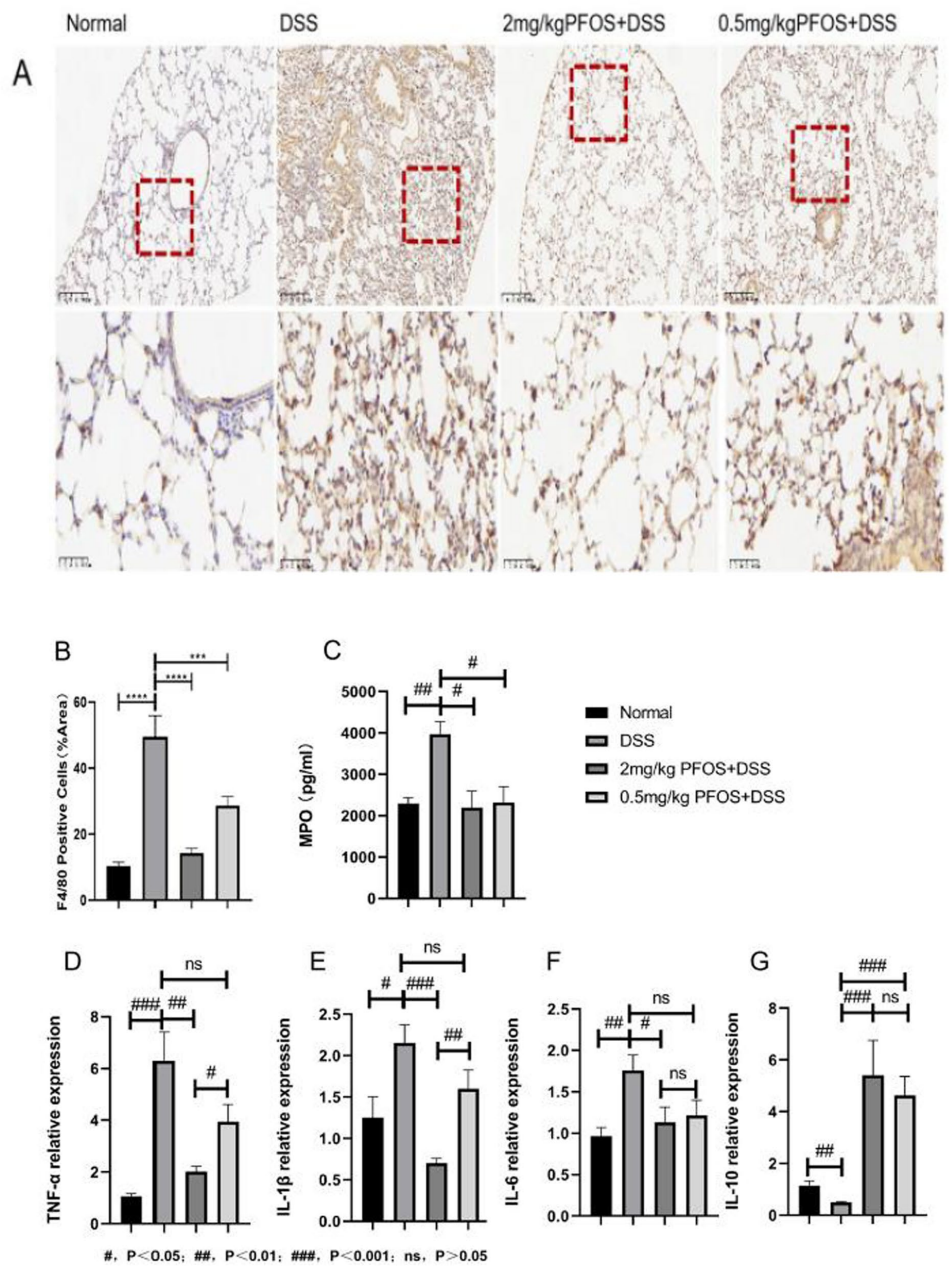
PFOS treatment reduced the inflammatory response in lung tissues of mice with colitis. **A**, **B** IHC staining images of macrophage-related protein F4/80 in lung tissues and immunohistochemical quantitative analysis of F4/80 (bar, 200 μm and 50 μm). **C** MPO levels in lung tissue (n = 5–6/group). **D**–**G** Impact of PFOS on inflammation-related gene expression (n = 5). Data are shown as the mean ± SEM. #p < 0.05, ##p < 0.01, ###p < 0.001

To assess the effect of PFOS on lung inflammation, we examined macrophage infiltration using F4/80 immunohistochemical staining. The DSS group exhibited significantly higher macrophage presence in the alveolar septum and peribronchus than the controls, which showed a marked reduced (Fig. 2A, B). Neutrophil accumulation, as indicated by elevated myeloperoxidase (MPO) activity in DSS-treated lung tissues, was significantly attenuated by PFOS (Fig. 2C). Additionally, qPCR analysis revealed that the DSS-induced upregulation of proinflammatory cytokines (IL-1β, IL-6, and TNF-α) in lung tissues was significantly suppressed by PFOS (2 mg/kg/d) (Fig. 2D, E). These results demonstrate that PFOS effectively mitigated lung inflammation in colitis-associated lung injury.

### PFOS reduces oxidative stress in lung tissues of colitis mice

**Fig. 3:**
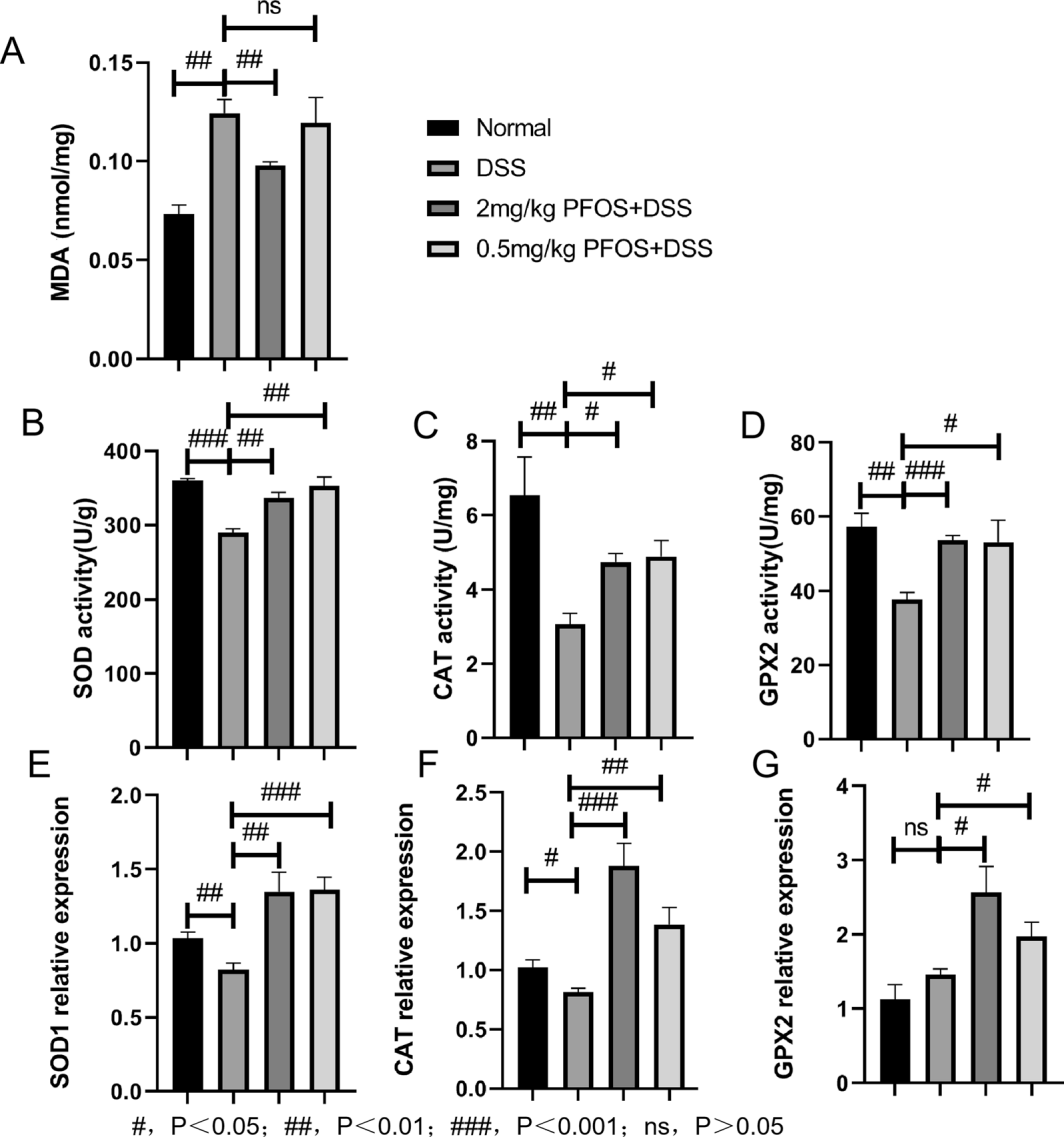
PFOS treatment reduces oxidative stress in the lung tissues of mice with colitis **A** Content of membrane lipid peroxidation (MDA) in lung tissue. **B**–**D** Changes in the activity of several typical antioxidant enzymes (SOD, GPX2, and CAT) (n = 5/each group). **E**–**G** Oxidative stress gene amelioration by PFOS analysis using qPCR (n = 5/each group). Data are shown as the mean ± SEM. #p < 0.05, ##p < 0.01, ###p < 0.001

Given the reported antioxidant effects of natural compounds via the Nrf2 pathway [21] and our previous findings on PFOS [11], we investigated PFOS’s ability of PFOS to counteract colitis-induced oxidative stress in lung tissues. Malondialdehyde (MDA) levels, a marker of oxidative stress, was significantly elevated in DSS-treated mice but was reduced by PFOS (2 mg/kg/d) (Fig. 3A). PFOS treatment (0.5 and 2 mg/kg/d) also restored the activity and mRNA expression of the antioxidant enzymes superoxide dismutase (SOD), catalase (CAT), and glutathione peroxidase 2 (GPX2) (Fig. 3B–G). These findings indicate that PFOS alleviates colitis-induced lung injury through its potent antioxidant effects.

### PFOS modulates NF-κB and Nrf2 signaling pathways

**Fig. 4:**
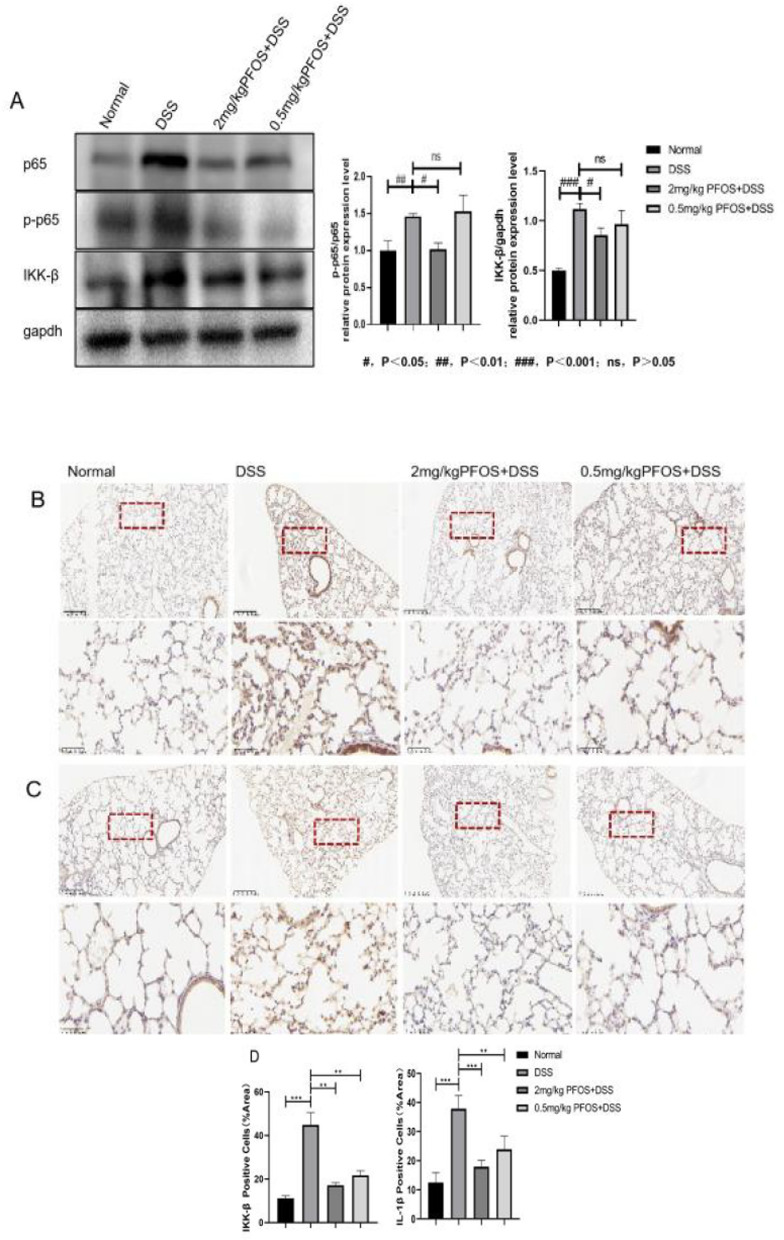

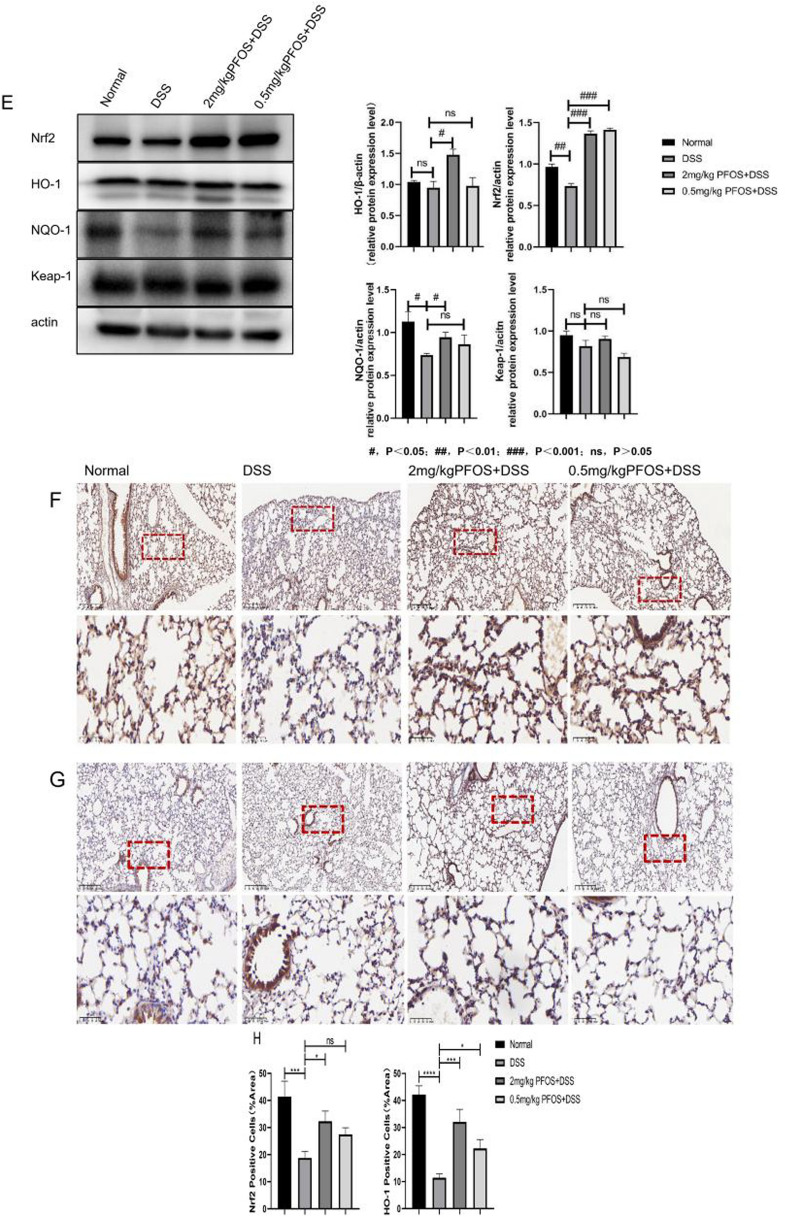
PFOS administration suppressed NF-kB signaling pathway and up regulated Nrf2 signaling pathway to reduce inflammation and oxidative stress **A** WB bands and relative protein expressions of NFkB signal path in lung tissues. (n ≥ 3/each group) **B**–**D** IHC staining images of IKK-β and IL-1β proteins in lung tissues (bar, 200 μm and 50 μm)(n = 5–6/each group), immunohistochemical quantitative analysis of IKK-β and IL-1β. **E** WB bands and relative protein expression of the Nrf2 signalling pathway in lung tissues. (n ≥ 3/each group). **F**–**H** Immunohistochemical staining images of Nrf2 and HO-1 proteins in lung tissues (bar, 200 μm and 50 μm)(n = 5–6/each group), immunohistochemical quantitative analysis of Nrf2 and HO-1. Data are shown as the mean ± SEM. #p < 0.05, ##p < 0.01, ###p < 0.001

NF-κB and Nrf2 pathways regulate inflammation and oxidative stress. We examined PFOS’s effects of PFOS on these pathways in DSS-treated mouse lung tissues. DSS administration increased p-p65/p65 ratio, while PFOS (2 mg/kg/d) significantly inhibited the expression of IKKβ and IL-1β, as confirmed by western blotting and immunohistochemistry (Fig. 4A–D). Conversely, DSS suppressed Nrf2, HO-1, and NQO-1 protein expression, which was restored by PFOS, with no significant changes in the Keap-1 levels (Fig. 4E–H). These results suggest that PFOS mitigates lung injury by suppressing NF-κB-mediated inflammation and enhancing Nrf2-mediated antioxidant response.

### PFOS inhibits LPS-induced damage in lung epithelial cells

**Fig. 5:**
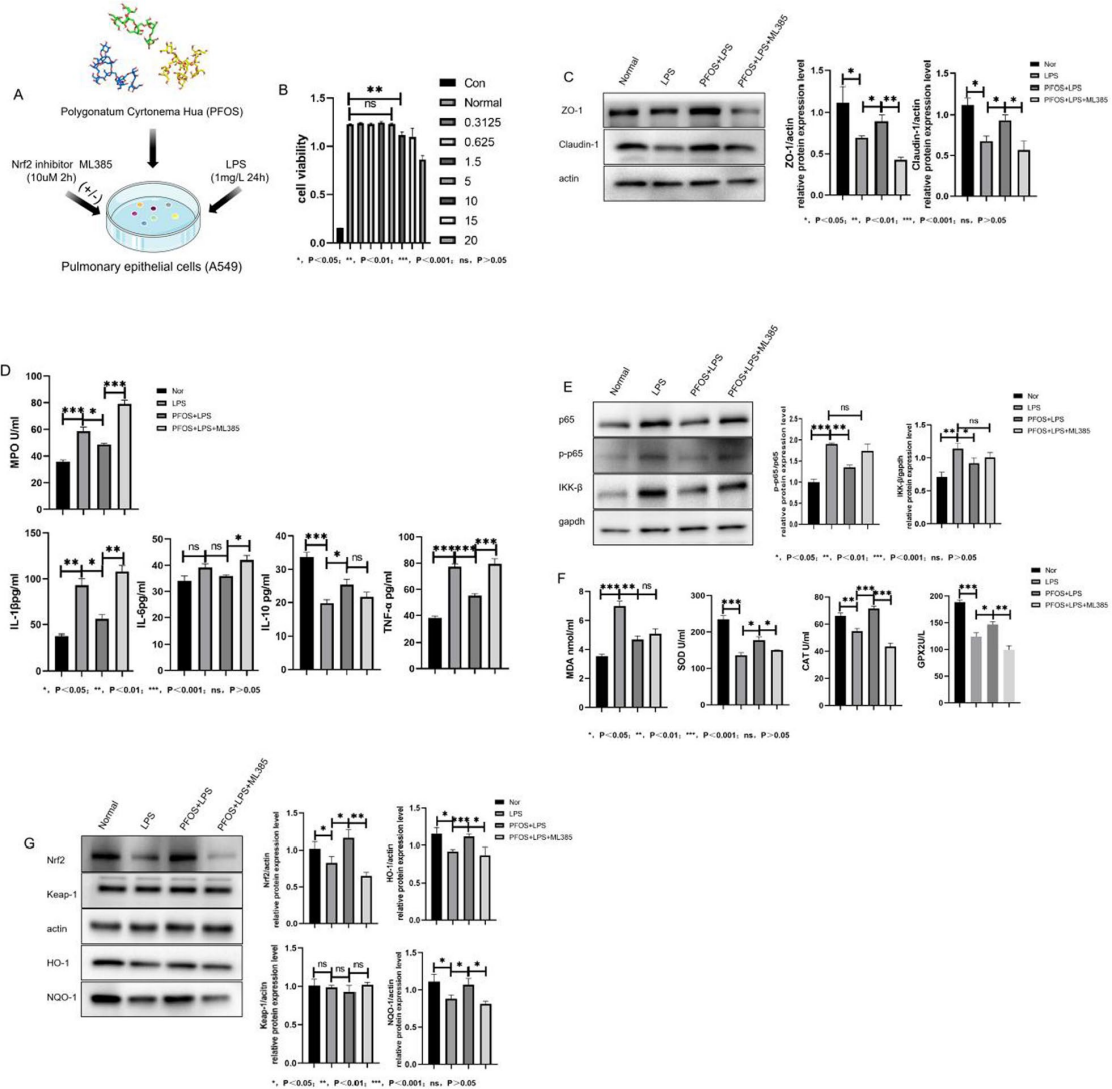
PFOS inhibits LPS-induced epithelial barrier damage, inflammation, and oxidative stress in lung epithelial cells (A549 cells). **A** Schematic diagram of PFOS treatment in LPS-induced A549 cells. **B** Cell viability was detected using CCK8 assay (n = 3). **C** WB bands and relative protein expression of epithelial barrier proteins (ZO-1 and Claudin-1) in cells. **D** MPO levels in the cell extract and TNF-α, IL-1β, and IL-6 levels in the supernatant. **E** WB bands and relative protein expression of the NF-κB signaling pathway. **F** Content of MDA and activity of several antioxidant enzymes in the cell extracts. **G** WB blotting and relative protein expression in the Nrf2 signaling pathway. Data are shown as the mean ± SEM. *p < 0.05, **p < 0.01, ***p < 0.001

To validate the mechanism of PFOS in vitro, we used LPS-stimulated A549 lung epithelial cells, which mimic inflammatory and oxidative stress conditions. The CCK8 assay identified 5 μM PFOS as a safe concentration because higher doses (10–20 μM) reduced cell viability (Fig. 5B). LPS reduced ZO-1 and Claudin-1 expression, which was partially restored by PFOS, an effect that was blocked by the Nrf2 inhibitor, ML385 (Fig. 5C). PFOS also reduced LPS-induced MPO, TNF-α, IL-1β, and IL-6 levels, and suppressed NF-κB signaling (p65, p-p65, and IKK-β) (Fig. 5D, E). While ML385 attenuated these effects, the changes were not statistically significant, suggesting limited direct Nrf2-NF-κB crosstalk (Fig. 5E). Additionally, PFOS counteracted LPS-induced reductions in SOD, GPX2, and CAT activity and increased MDA levels, which were abrogated by ML385 (Fig. 5F). Western blot analysis confirmed that PFOS restored the expression of Nrf2, HO-1, and NQO-1 expression (Fig. 5G). These data indicate that PFOS mitigated LPS-induced epithelial barrier damage, inflammation, and oxidative stress via NF-κB inhibition and Nrf2 activation.

### PFOS modulates intestinal metabolites linked to lung inflammation

**Fig. 6:**
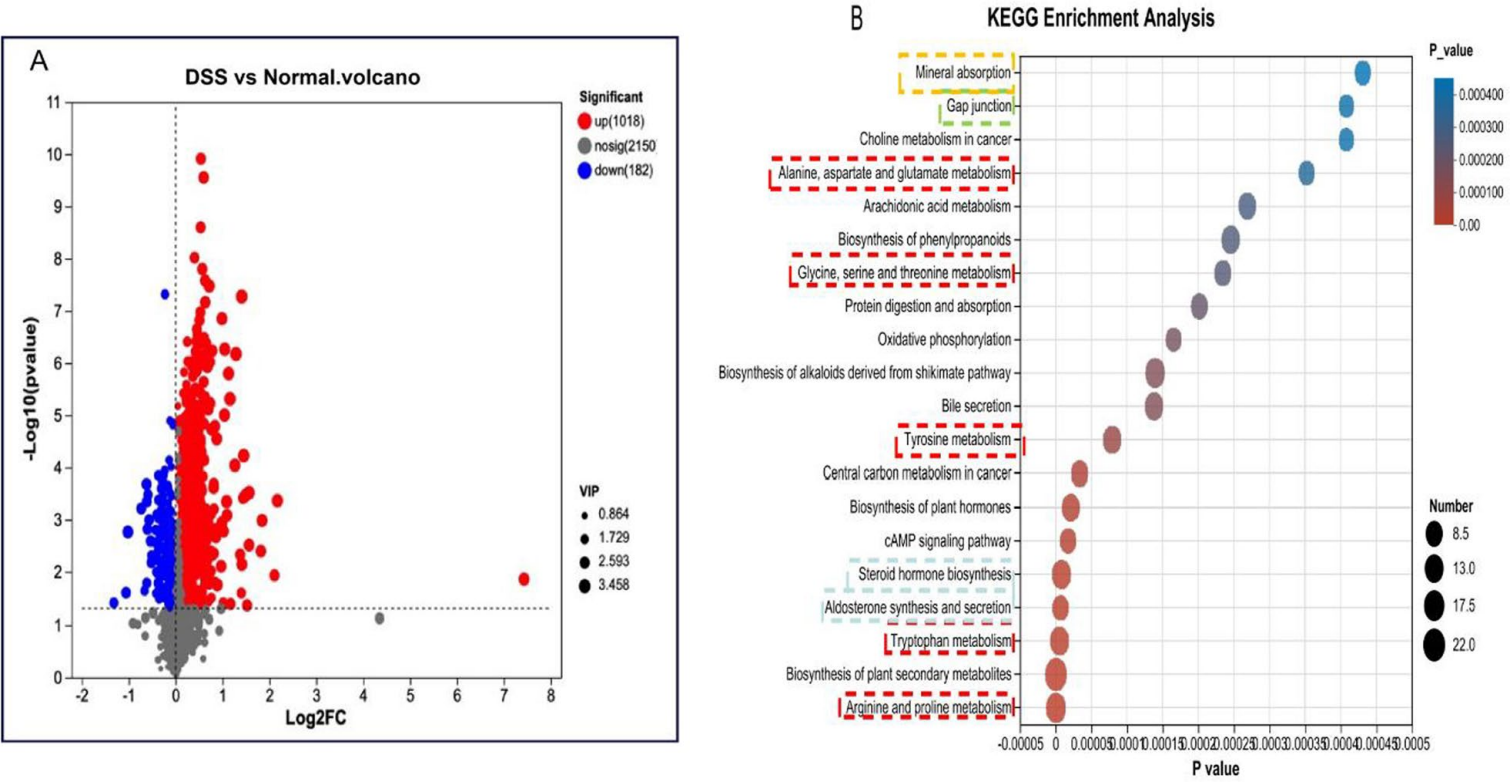
Analysis of different metabolites between DSS and normal control groups. **A** represents the volcanic map of the different metabolites compared between the two groups, the blue dot indicates the difference in metabolites downregulated, the red dot indicates the difference in metabolites upregulated, the more the left and right sides and the upper point the difference in expression is more significant; **B** represents KEGG pathway enrichment analysis of metabolites in the normal control group was significantly different from the DSS model group(the red and blue boxes indicate amino acid and lipid metabolic pathways, respectively)

**Fig. 7:**
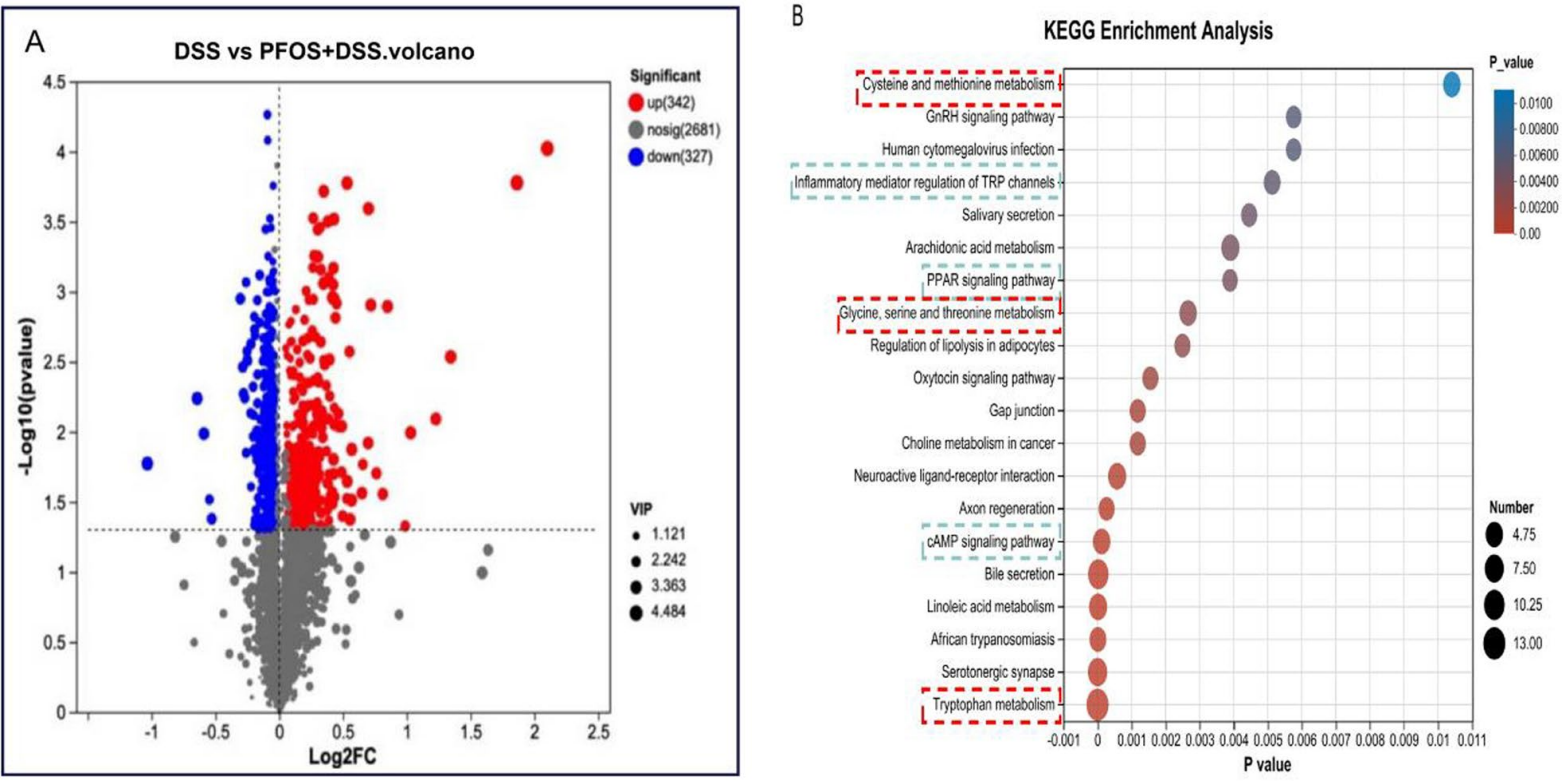
Multivariate statistical analysis of fecal metabolites in the PFOS pre-intervention and DSS model groups. **A** represents the volcanic map of the different metabolites compared between the two groups, the blue dot indicates the difference in metabolites downregulated, the red dot indicates the difference in metabolites upregulated, the more the left and right sides and the upper point the difference in expression is more significant; **B** represents KEGG pathway enrichment analysis of metabolites in the PFOS preintervention group was significantly different from the DSS model group (the red and blue boxes indicate amino acid metabolic pathways and inflammatory regulatory pathways, respectively)

**Fig. 8:**
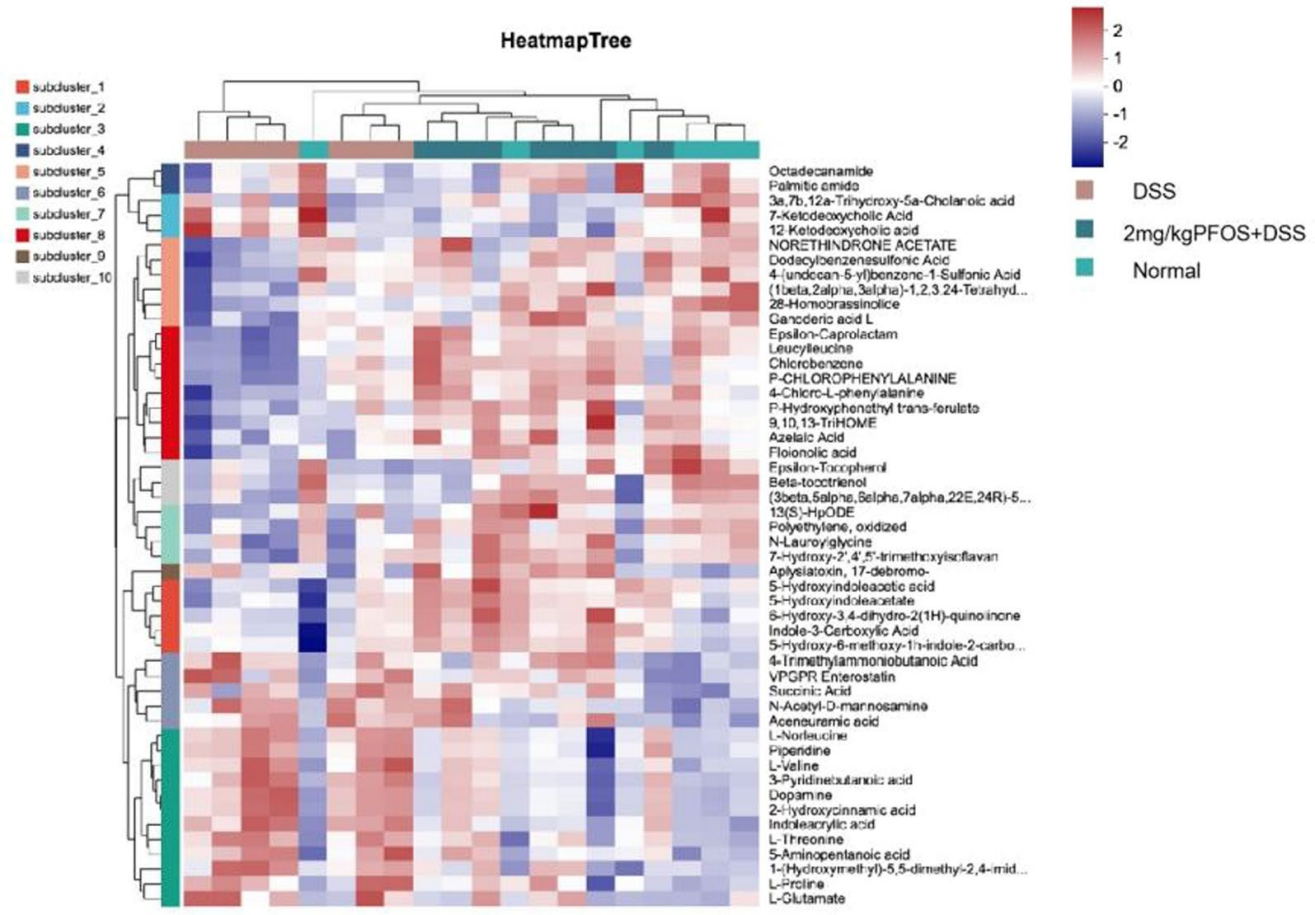
The two metabolites were combined and analyzed by clustering different metabolites

Our previous studies have shown that oligosaccharides restore gut microbiota imbalances in DSS-induced enteritis [9]. Here, non-targeted fecal metabolomics revealed that PFOS pre-intervention shifted metabolite profiles closer to those of the controls (Supplementary Fig. 1A-B). Orthogonal partial least squares discriminant analysis (OPLS-DA) identified the differential metabolites (p < 0.05, VIP > 1.0) (Fig. 6A and Supplementary Fig. 2). KEGG pathway analysis indicated DSS-induced disruptions in amino acid and lipid metabolism, which were ameliorated by PFOS (Figs. 6B, 7A and Supplementary Fig. 3). Supplementary Table S2A showed that the significantly different metabolites in the normal control group and the DSS model group were annotated by KEGG and classified at the KEGG compound level, totaling 54 differentially expressed metabolites (the screening criteria were VIP ≥ 1, P < 0.05). Notably, PFOS enriched metabolites in the cAMP, PPAR, and TRP pathways, which are known regulators of NF-κB and Nrf2 signaling [22] (Fig. 7B). Supplementary Table S2B shows that 24 significantly different metabolites that showed significant differences between the PFOS pre-intervention experimental group and the DSS model group were annotated by KEGG, and they were classified at the KEGG compound level (the screening condition was VIP ≥ 1, P < 0.05).Cluster analysis and heatmaps confirmed distinct metabolic profiles in the DSS group compared with similar profiles in the PFOS-treated and control groups (Fig. 8). These findings suggest that PFOS mitigates lung inflammation by modulating intestinal metabolites linked to the inflammatory and oxidative pathways.

## Discussion

Ulcerative colitis is a systemic inflammatory disorder associated with extraintestinal manifestations, including low-grade pulmonary inflammation in nearly half of patients [23]. The gut-lung axis facilitates bidirectional communication through microbial metabolites, mucosal barrier dysfunction, and immune cell homing [24]. Using a dextran sodium sulfate (DSS)-induced enteritis mouse model, we confirmed lung tissue damage, consistent with prior studies [25]. Pre-administration of *Polygonatum cyrtonema* oligosaccharides (PFOS), previously shown to mitigate intestinal and pulmonary inflammation [10, 11], significantly reduced lung injury scores, particularly at higher doses (Fig. 1B). PFOS restored the expression and membrane organization of tight junction proteins ZO-1 and Claudin-1, which are critical for epithelial barrier integrity [26], reducing cytoplasmic droplet accumulation in DSS-treated mice (Fig. 1C–E). In vitro, PFOS similarly enhanced ZO-1 and Claudin-1 expression in LPS-stimulated A549 lung epithelial cells (Fig. 5C), suggesting that PFOS promotes tight junction formation, potentially via protein phase separation, thereby mitigating epithelial barrier dysfunction and the associated lung injury.

Our previous work demonstrated that PFOS effectively alleviates DSS-induced colitis by reducing colonic inflammation, oxidative stress, and epithelial barrier damage [11]. Building upon these findings, the present study extends the protective role of PFOS to extra-intestinal organ injury, specifically the lung. Notably, lung inflammation and oxidative stress were significantly attenuated in PFOS-treated mice, despite the lung not being a direct target of DSS exposure.

The parallel improvement in colonic pathology (as previously reported) and pulmonary outcomes (as demonstrated in the current study) supports a gut–lung axis mechanism, whereby intestinal inflammation acts as a driver of systemic and pulmonary inflammatory responses. PFOS-mediated suppression of colonic inflammation and oxidative stress likely reduces circulating inflammatory mediators and oxidative burden, thereby indirectly protecting lung tissue. While direct causal links were not experimentally dissected in the present study, the consistency between intestinal and pulmonary protective effects provides strong associative evidence supporting this mechanistic framework.

Inflammation is a key driver of epithelial barrier compromise in both the gut and lungs, enabling the systemic translocation of bacterial toxins such as lipopolysaccharide (LPS), which exacerbates distant organ inflammation. In the DSS model, lung tissues exhibited elevated macrophage (F4/80) and neutrophil (MPO) infiltration, and PFOS (2 mg/kg/d) significantly reduced (Fig. 2A–C). PFOS also suppressed the mRNA expression of pro-inflammatory cytokines (IL-1β, IL-6, and TNF-α) in lung tissues and the protein levels in LPS-treated A549 cells (Figs. 2D–G, 5D). Given the central role of NF-κB in the activation of inflammatory mediator activation [27], we investigated its involvement in PFOS. PFOS markedly reduced IKKβ, p65, and phosphorylated p65 expression, and inhibited NF-κB signaling in both in vivo and in vitro models (Figs. 4A, 5E). These findings indicate that PFOS attenuates lung inflammation in enteritis-associated injury by downregulating the NF-κB/IKK signaling pathway.

Oxidative stress, driven by excessive reactive oxygen species (ROS), exacerbates proinflammatory signaling in lung pathology [28]. PFOS treatment significantly reduced malondialdehyde (MDA) levels, a lipid peroxidation marker, while enhancing the activity and expression of the antioxidant enzymes superoxide dismutase (SOD), catalase (CAT), and glutathione peroxidase 2 (GPX2) in both mouse lung tissues and A549 cells (Figs. 3, 5F). Nuclear factor erythroid 2-related factor 2 (Nrf2), a master regulator of antioxidant defenses [29], was upregulated by PFOS, along with its downstream targets heme oxygenase-1 (HO-1) and NAD(P)H quinone dehydrogenase 1 (NQO-1), independently of Keap1 modulation (Figs. 4E–H, 5G). This Keap1-independent Nrf2 activation is consistent with reports on alternative regulatory mechanisms involving protein kinases [30]. Furthermore, crosstalk between the Nrf2 and NF-κB pathways, where Nrf2 suppresses NF-κB activity [31], was explored using the Nrf2 inhibitor ML385. The partial abrogation of PFOS’s anti-inflammatory effects of PFOS by ML385 suggests indirect Nrf2-NF-κB interactions, reinforcing PFOS’s protective role of PFOS via Nrf2 activation (Fig. 5).

Intestinal metabolites are increasingly recognized as modulators of the lung immune response [32]. Non-targeted fecal metabolomics revealed DSS-induced disruptions in amino acid and lipid metabolism, which PFOS intervention significantly ameliorated by PFOS intervention (Figs. 6B, 7A and Supplementary Fig. 3). Notably, PFOS enriched metabolites in the cAMP, PPAR, and TRP pathways, which are known regulators of NF-κB and Nrf2 signaling (Fig. 7B). Cluster analysis confirmed that PFOS restored metabolic profiles closer to those of the controls (Fig. 8), consistent with previously reported prebiotic effects on gut microbiota [9]. These research findings indicated that PFOS may influence the cAMP, PPAR, and TRP pathways by regulating intestinal metabolites, thereby participating in the regulation of NF-κB and Nrf2 signaling, alleviating enteritis-associated lung injury by modulating the gut-lung axis.

## Conclusion

**Fig. 9:**
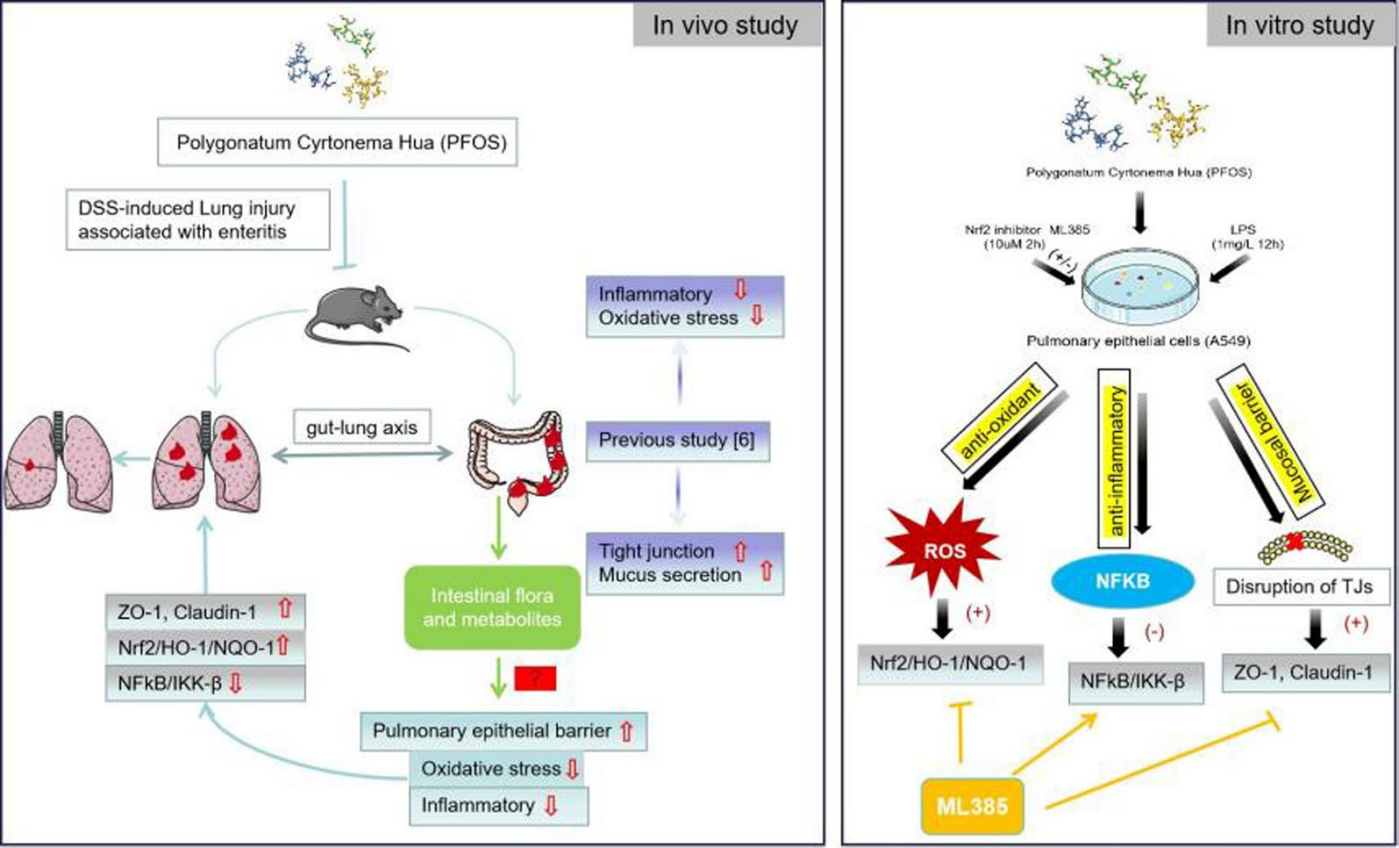
Efficacy and mechanism of PFOS in lung inflammation and epithelial barrier damage in the treatment of enteritis-associated lung injury

This study demonstrated that PFOS mitigated enteritis-associated lung injury by enhancing epithelial barrier integrity and modulating the Nrf2/NF-κB signaling pathway (Fig. 9). PFOS exerts robust anti-inflammatory and antioxidant effects by upregulating antioxidant proteins (Nrf2, HO-1, and NQO-1) and suppressing proinflammatory markers (p65, p-p65, and IKKβ). Modulation of intestinal metabolites further supports their protective role against lung inflammation. These findings suggest that PFOS is a promising therapeutic candidate for the management of lung injuries associated with intestinal inflammation.

However, this study primarily relied on animal models and in vitro cellular systems, both of which have inherent limitations. Especially, the A549 cells selected for this study belong to a human lung adenocarcinoma epithelial cell line, exhibited chromosomal abnormalities and disrupted signaling pathways. Their inflammatory response is different from that of normal primary lung alveolar epithelial cells. Therefore, future studies are warranted to validate these findings in more cell models, such as using primary alveolar epithelial cells or alveolar epithelial cells differentiated from induced pluripotent stem cells.The application of Nrf2 inhibitors (ML385) in animal models is also necessary.

## Supplementary Information

Supplementary material 1.

Supplementary material 2.

## Data Availability

Data supporting the findings of this study are available upon request from the corresponding author.

## Abbreviations

UC: Ulcerative colitis
PFOS: Oligosaccharides from *Polygonatum cyrtonema* Hua
DSS: Dextran sodium sulfate
LPS: Lipopolysaccharide
IBD: Inflammatory bowel disease
Nrf2: Nuclear factor-erythroid 2-related factor 2
ROS: Reactive oxygen species
COPD: Chronic obstructive pulmonary disease
GPx: Glutathione peroxidase
GSH: Glutathione
HO-1: Heme oxygenase-1
H&E: Hematoxylin and eosin
SDS-PAGE: Sodium dodecyl sulfate polyacrylamide gel electrophoresis
PVDF: Polyvinylidene difluoride membranes
HRP: Horseradish peroxidase
ALI: Acute lung injury
MPO: Myeloperoxidase
MDA: Malondialdehyde
PCA: Principal component analysis
OPLS-DA: Orthogonal partial least squares discriminant analysis
KEGG: Kyoto Encyclopedia of Genes and Genomes
IKK: IκB kinase; p-p65, phosphorylated p65
NQO-1: NAD(P)H quinone dehydrogenase 1
PPAR: Peroxisome proliferator-activated receptor
TRP: Transient receptor potential

## References

[1] Kröner PT, Lee A, Farraye FA. Respiratory tract manifestations of inflammatory bowel disease. Inflamm Bowel Dis. 2021;27(4):563–74. 10.1093/ibd/izaa112. 32448912 10.1093/ibd/izaa112

[2] Camus P, Colby TV. Spectrum of airway involvement in inflammatory bowel disease. Clin Chest Med. 2022;43(1):141–55. 10.1016/j.ccm.2021.12.003. 35236554 10.1016/j.ccm.2021.12.003

[3] Wang L, Cai Y, Garssen J, et al. The bidirectional gut-lung axis in chronic obstructive pulmonary disease. Am J Respir Crit Care Med. 2023;207(9):1145–60. 10.1164/rccm.202206-1066TR. 36883945 10.1164/rccm.202206-1066TR PMC10161745

[4] He Q, Shi Y, Tang Q, et al. Herbal medicine in the treatment of COVID-19 based on the gut-lung axis. Acupunct Herb Med. 2022;2(3):172–83. 10.1097/HM9.0000000000000038. 37808350 10.1097/HM9.0000000000000038 PMC9746256

[5] Zhang J-J, Dong X, Cao Y-Y, et al. Clinical characteristics of 140 patients infected with SARS-CoV-2 in Wuhan, China. Allergy. 2020;75(7):1730–41. 10.1111/all.14238. 32077115 10.1111/all.14238

[6] Kisiel MA, Sedvall M, Malinovschi A, et al. Inflammatory bowel disease and asthma. Results from the RHINE study. Respir Med. 2023;216:107307. 10.1016/j.rmed.2023.107307. 37271300 10.1016/j.rmed.2023.107307

[7] Lou Z, Zhao H, Lyu G. Mechanism and intervention of mucosal immune regulation based on “lung and large intestine being interior-exteriorly related” theory of traditional Chinese medicine. Zhejiang Da Xue Xue Bao Yi Xue Ban. 2020;49(6):665–78. 10.3785/j.issn.1008-9292.2020.12.01. 33448169 10.3785/j.issn.1008-9292.2020.12.01 PMC8800704

[8] Zhao P, Zhao C, Li X, et al. The genus *Polygonatum*: a review of ethnopharmacology, phytochemistry, and pharmacology. J Ethnopharmacol. 2018;214:274–91. 10.1016/j.jep.2017.12.006. 29246502 10.1016/j.jep.2017.12.006

[9] Li XL, Ma RH, Zhang F, et al. Evolutionary research trend of *Polygonatum* species: a comprehensive account of their transformation from traditional medicines to functional foods. Crit Rev Food Sci Nutr. 2023;63(19):3803–20. 10.1080/10408398.2021.1993783. 34669530 10.1080/10408398.2021.1993783

[10] He L, Yan B, Yao C, et al. Oligosaccharides from *PolygonatumCyrtonemaHua*: structural characterization and treatment of LPS-induced peritonitis in mice. Carbohydr Polym. 2021;255:117392. 10.1016/j.carbpol.2020.117392. 33436221 10.1016/j.carbpol.2020.117392

[11] Xu J, Tang C, Din AU, et al. Oligosaccharides of *PolygonatumCyrtonemaHua* ameliorates dextran sulfate sodium-induced colitis and regulates the gut microbiota. Biomed Pharmacother. 2023;161:114562. 10.1016/j.biopha.2023.114562. 36934554 10.1016/j.biopha.2023.114562

[12] Raftery AL, Caitlin A, et al. Development of severe colitis is associated with lung inflammation and pathology. Front Immunol. 2023;31:1125260. 10.3389/fimmu.2023.1125260. 10.3389/fimmu.2023.1125260 PMC10102339 37063825

[13] Li Y, Cao Y, Xiao J, et al. Inhibitor of apoptosis-stimulating protein of p53 inhibits ferroptosis and alleviates intestinal ischemia/reperfusion-induced acute lung injury. Cell Death Differ. 2020;27(9):2635–50. 10.1038/s41418-020-0528-x. 32203170 10.1038/s41418-020-0528-x PMC7429834

[14] Halliwell B. Understanding mechanisms of antioxidant action in health and disease. Nat Rev Mol Cell Biol. 2024;25(1):13–33. 10.1038/s41580-023-00645-4. 37714962 10.1038/s41580-023-00645-4

[15] Dong H, Qiang Z, Chai D, et al. Nrf2 inhibits ferroptosis and protects against acute lung injury due to intestinal ischemia reperfusion via regulating *SLC7A11* and *HO-1*. Aging. 2020;12(13):12943–59. 10.18632/aging.103378. 32601262 10.18632/aging.103378 PMC7377827

[16] Battino M, Giampieri F, Pistollato F, et al. Nrf2 as regulator of innate immunity: a molecular Swiss army knife! Biotechnol Adv. 2018;36(2):358–70. 10.1016/j.biotechadv.2017.12.012. 29277308 10.1016/j.biotechadv.2017.12.012

[17] Qin T, Du R, Huang F, et al. Sinomenine activation of Nrf2 signaling prevents hyperactive inflammation and kidney injury in a mouse model of obstructive nephropathy. Free Radic Biol Med. 2016;92:90–9. 10.1016/j.freeradbiomed.2016.01.011. 26795599 10.1016/j.freeradbiomed.2016.01.011

[18] Smith KM, Mrozek JD, Simonton SC, et al. Prolonged partial liquid ventilation using conventional and high-frequency ventilatory techniques: gas exchange and lung pathology in an animal model of respiratory distress syndrome. Crit Care Med. 1997;25(11):1888–97. 10.1097/00003246-199711000-00030. 9366775 10.1097/00003246-199711000-00030

[19] Ge P, Luo Y, Okoye CS, et al. Intestinal barrier damage, systemic inflammatory response syndrome, and acute lung injury: a troublesome trio for acute pancreatitis. Biomed Pharmacother. 2020;132:110770. 33011613 10.1016/j.biopha.2020.110770

[20] Beutel O, Maraspini R, Pombo-García K, et al. Phase separation of Zonula occludens proteins drives formation of tight junctions. Cell. 2019;179(4):923-936.e11. 10.1016/j.cell.2019.10.011. 31675499 10.1016/j.cell.2019.10.011

[21] Luan R, Ding D, Yang J. The protective effect of natural medicines against excessive inflammation and oxidative stress in acute lung injury by regulating the Nrf2 signaling pathway. Front Pharmacol. 2022;13:1039022. 36467050 10.3389/fphar.2022.1039022 PMC9709415

[22] Zhong CC, Zhao T, Hogstrand C, et al. Copper (Cu) induced changes of lipid metabolism through oxidative stress-mediated autophagy and Nrf2/PPARgamma pathways. J Nutr Biochem. 2022;100:108883. 10.1016/j.jnutbio.2021.108883. 34653601 10.1016/j.jnutbio.2021.108883

[23] Mohamed-Hussein AA, Mohamed NA, Ibrahim ME. Changes in pulmonary function in patients with ulcerative colitis. Respir. 2007;101:977–82. 10.1016/j.rmed.2006.09.005. 10.1016/j.rmed.2006.09.005 17049827

[24] Schmid F, Chao CM, Däbritz J. Pathophysiological concepts and management of pulmonary manifestation of pediatric inflammatory bowel disease. Int J Mol Sci. 2022;23(13):7287. 10.3390/ijms23137287. 35806292 10.3390/ijms23137287 PMC9266732

[25] Aydin B, Songur Y, Songur N, et al. Investigation of pulmonary involvement in inflammatory bowel disease in an experimental model of colitis. Korean J Intern Med. 2016;31(5):853–9. 10.3904/kjim.2014.238. 27539446 10.3904/kjim.2014.238 PMC5016269

[26] Akdis CA. Does the epithelial barrier hypothesis explain the increase in allergy, autoimmunity, and other chronic conditions? Nat Rev Immunol. 2021;21(11):739–51. 10.1038/s41577-021-00538-7. 33846604 10.1038/s41577-021-00538-7

[27] Liu T, Zhang L, Dongyun J, et al. NF-kB signaling in inflammation. Signal Transduct Target Ther. 2017;2:e17023. 10.1038/sigtrans.2017.23 PMC5661633 29158945

[28] Xu W, Zhao T, Xiao H. The implication of oxidative stress and AMPK-Nrf2 antioxidative signaling in pneumonia pathogenesis. Front Endocrinol (Lausanne). 2020;11:400. 10.3389/fendo.2020.00400. 32625169 10.3389/fendo.2020.00400 PMC7311749

[29] Tonelli C, Chio IIC, Tuveson DA. Transcriptional Regulation by Nrf2. Antioxid Redox Signal. 2018;29(17):1727–45. 10.1089/ars.2017.7342. 28899199 10.1089/ars.2017.7342 PMC6208165

[30] Peng S, Shen L, Yu X, et al. The role of Nrf2 in the pathogenesis and treatment of ulcerative colitis. Front Immunol. 2023;5(14):1200111. 10.3389/fimmu.2023.1200111. 10.3389/fimmu.2023.1200111 PMC10285877 37359553

[31] Casper E. The crosstalk between Nrf2 and NF-κB pathways in coronary artery disease: can it be regulated by SIRT6? Life Sci. 2023;330:122007. 10.1016/j.lfs.2023.122007. 37544377 10.1016/j.lfs.2023.122007

[32] Ma PJ, Wang MM, Wang Y. Gut microbiota: a new insight into lung diseases. Biomed Pharmacother. 2022;155:113810. 10.1016/j.biopha.2022.113810. 36271581 10.1016/j.biopha.2022.113810

