# Supplementary material for: Oligosaccharides from Polygonatum cyrtonema Hua ameliorate colitis-induced lung injury via modulation of the gut-lung axis through NF-κB and Nrf2 pathways
Source: Nat Prod Bioprospect. 2026 Jun 23;16(1):70. doi: 10.1007/s13659-026-00608-0 (PMC13291371; doi:10.1007/s13659-026-00608-0)
Supplement: Supplementary file 1 — Supplementary material 1. [file 13659_2026_608_MOESM1_ESM.pdf]

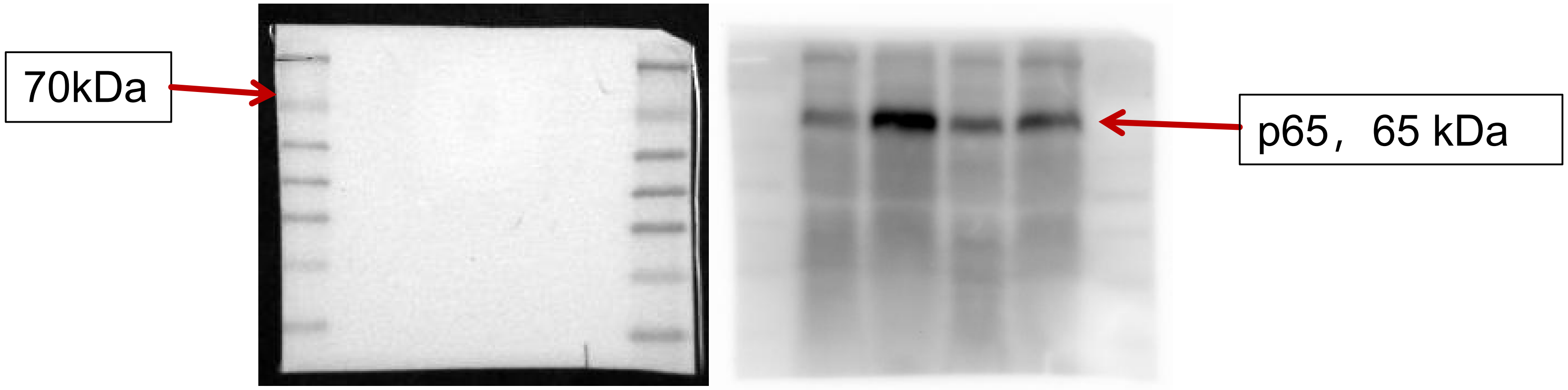

Figure 4A P65 is the key component of NFkB signal path, p65 expression was assessed by Westernblotting in lung tissues from Normal control group, DSS model group, 2mg/kg PFOS intervention group, 0.5mg/kg PFOS intervention group.

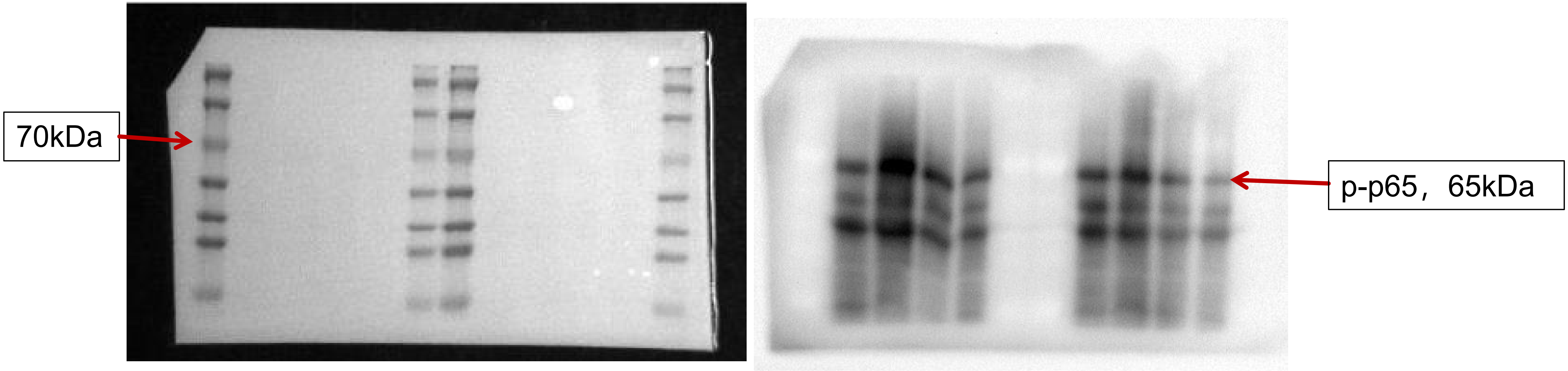

Figure 4A p-p65 is an other key component of NFkB signal path, p-p65 expression was assessed by Westernblotting in lung tissues from Normal control group, DSS model group, 2mg/kg PFOS intervention group, 0.5mg/kg PFOS intervention group.

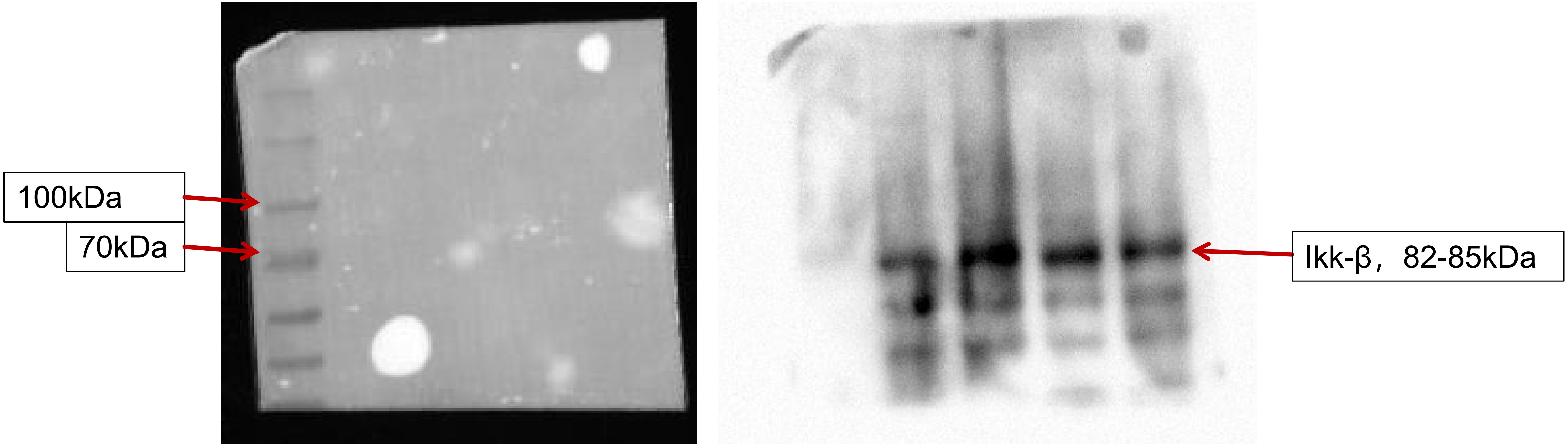

Figure 4A Ikk-β is an other key component of NFkB signal path, Ikk-β expression was assessed by Westernblotting in lung tissues from Normal control group, DSS model group, 2mg/kg PFOS intervention group, 0.5mg/kg PFOS intervention group.

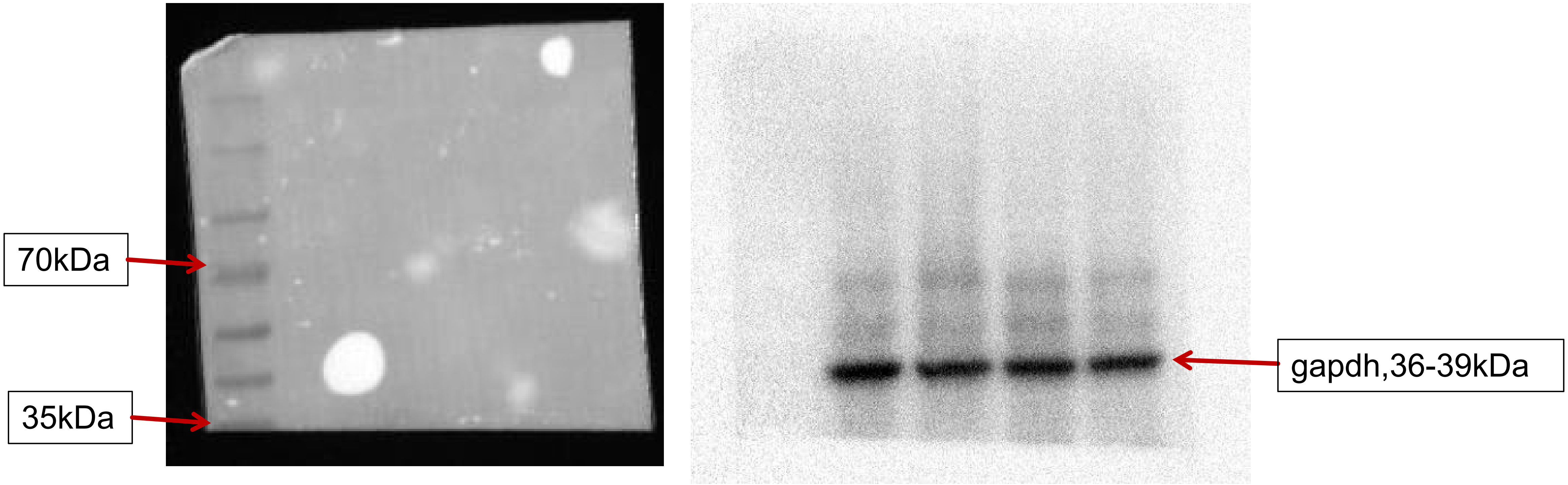

Figure 4A The molecular weight of Gapdh is 36-39 kDa.

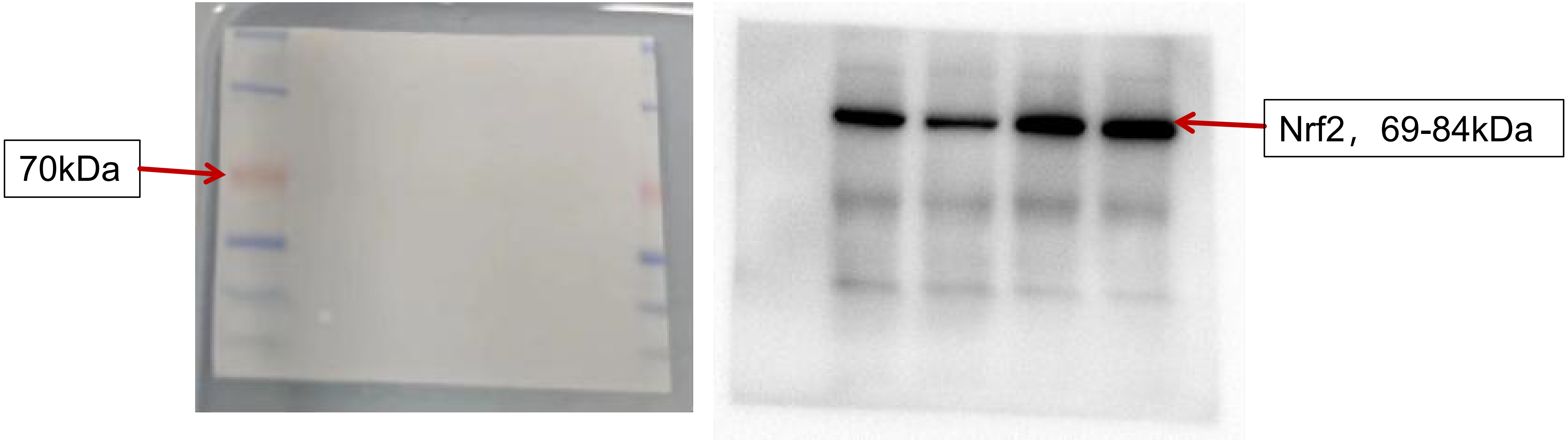

Figure 4D Nrf2 is a key protein in the oxidative stress signaling pathway, Nrf2 expression was assessed by Westernblotting in lung tissues from Normal control group, DSS model group, 2mg/kg PFOS intervention group, 0.5mg/kg PFOS intervention group.

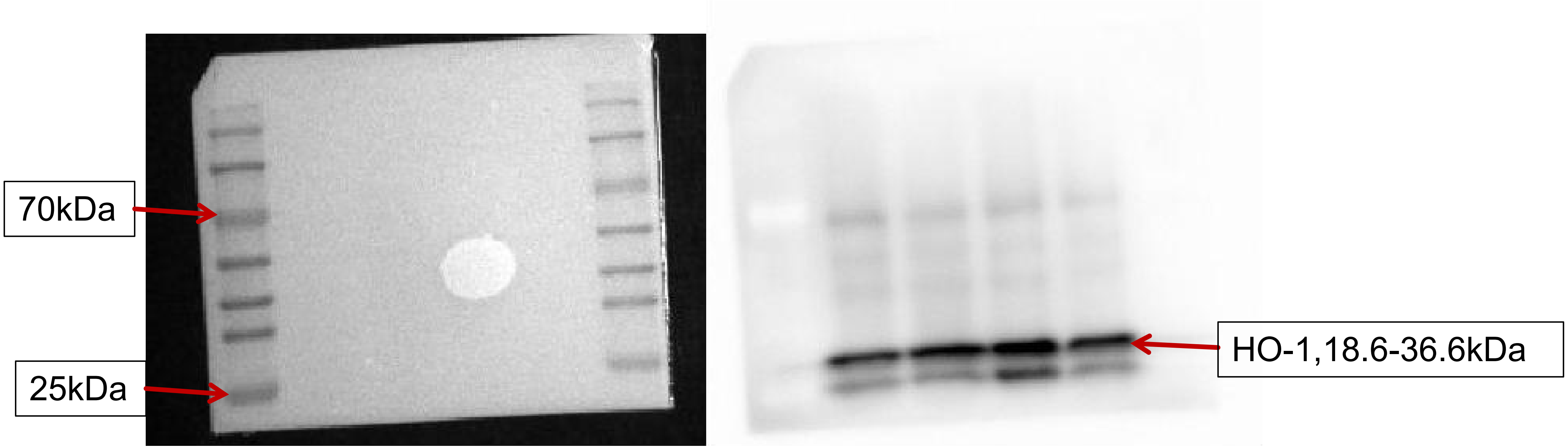

Figure 4D HO-1 is a key protein in the oxidative stress signaling pathway, HO-1 expression was assessed by Westernblotting in lung tissues from Normal control group, DSS model group, 2mg/kg PFOS intervention group, 0.5mg/kg PFOS intervention group.

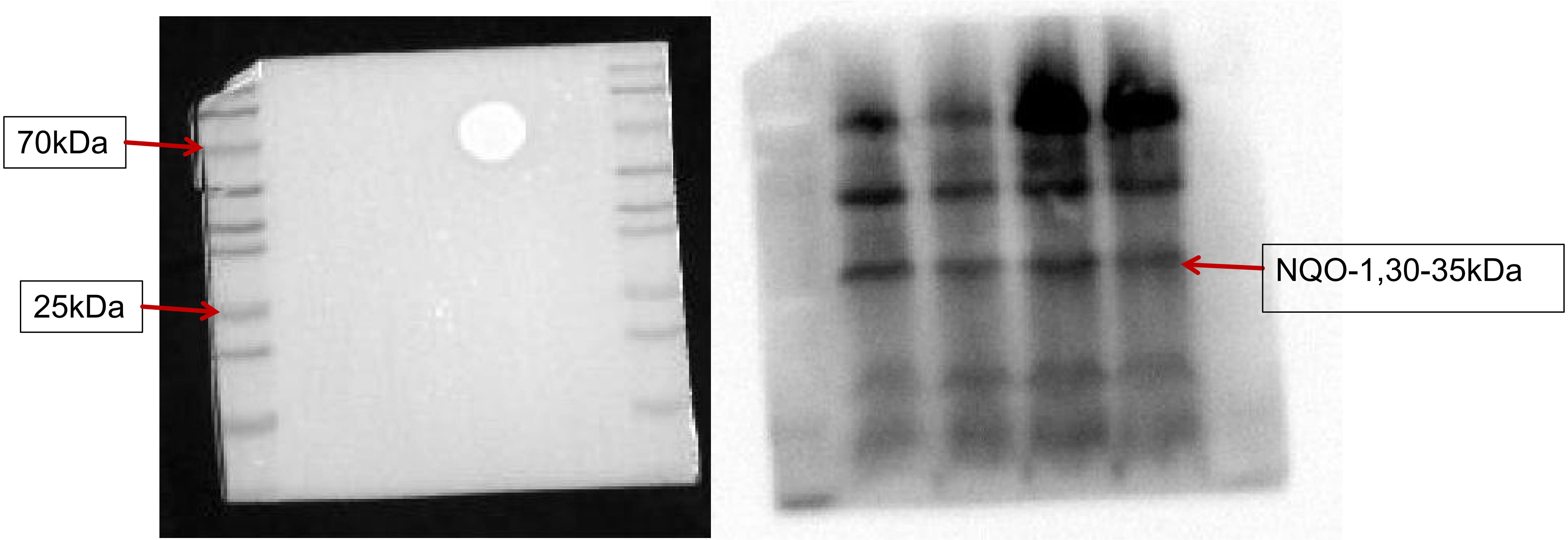

Figure 4D NQO-1 is a key protein in the oxidative stress signaling pathway, NQO-1 expression was assessed by Westernblotting in lung tissues from Normal control group, DSS model group, 2mg/kg PFOS intervention group, 0.5mg/kg PFOS intervention group.The molecular weight of NQO-1 is 30-35 kDa.

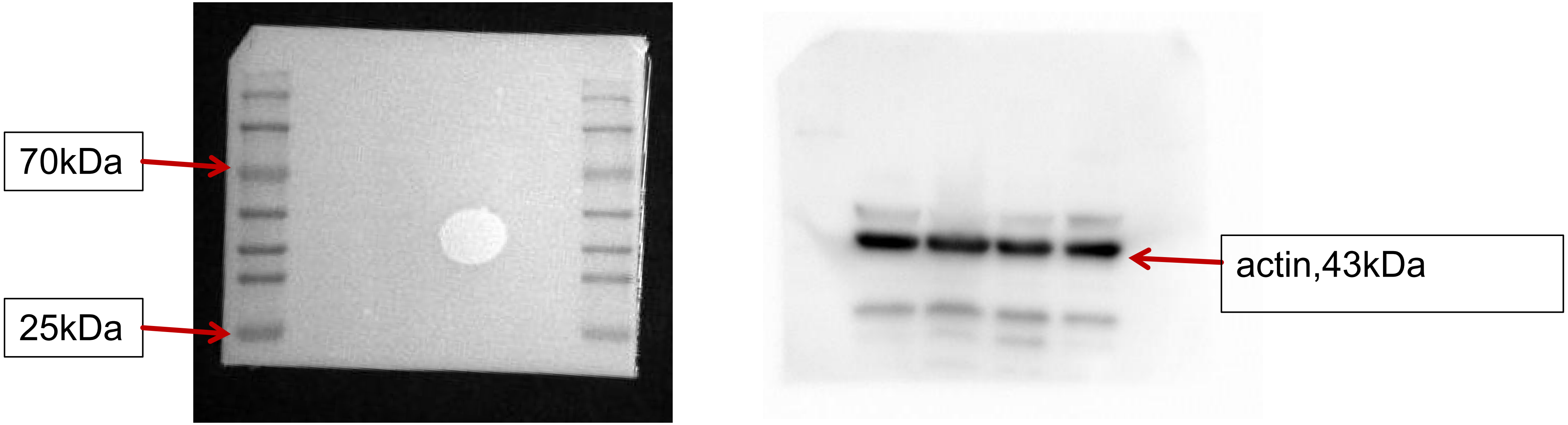

Figure 4D The molecular weight of actin is 43kDa.

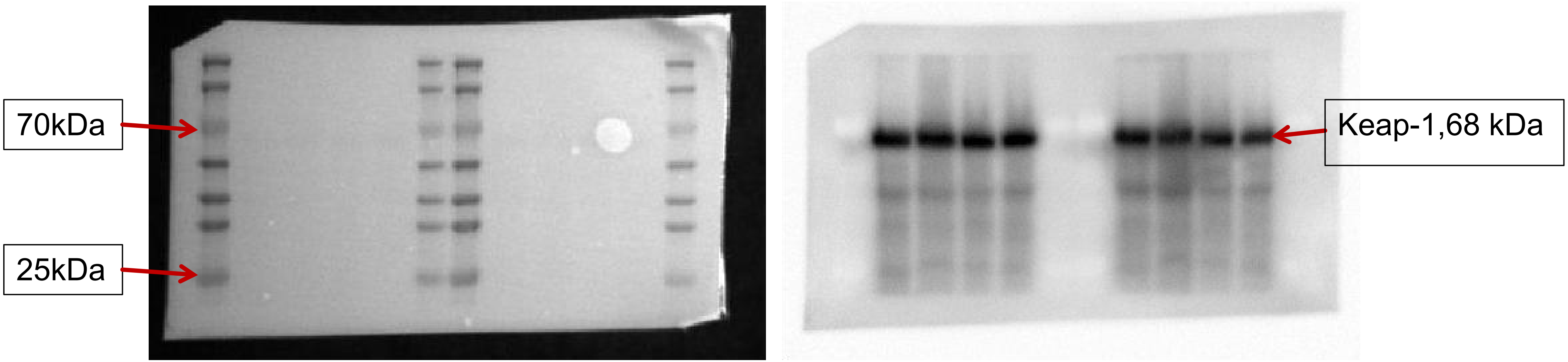

Figure 4D Keap-1 is a key protein in the oxidative stress signaling pathway.The molecular weight of Keap-1 is 68 kDa.

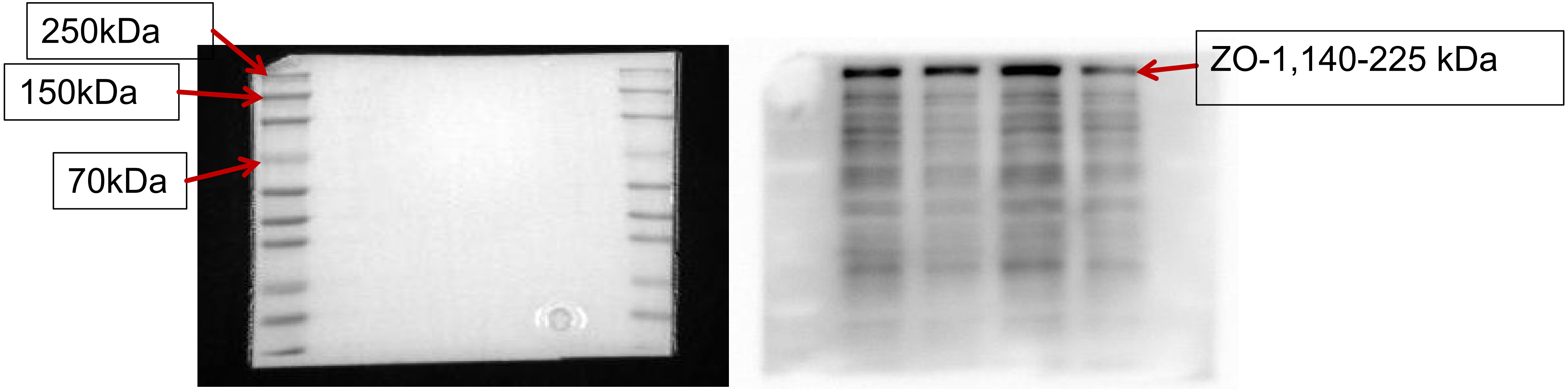

Figure 5C ZO-1, one of the core components of tight junctions. ZO-1 expression was assessed by Westernblotting in A549 lung epithelial cells from Normal control group, LPS intervention group, PFOS intervention group, Nrf2 Inhibitor ML385 intervention group.

70kDa

25kDa

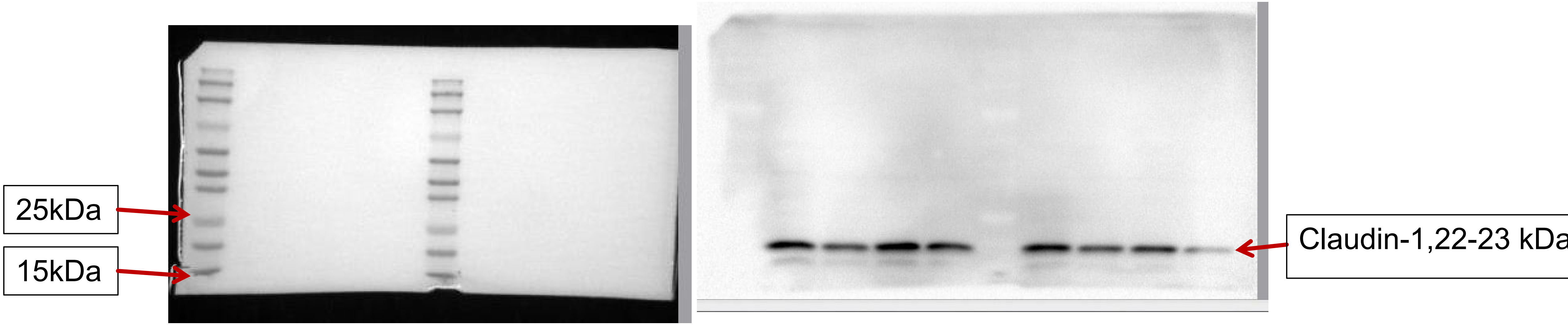

Figure 5C Claudin-1, one of the core components of tight junctions. Claudin-1 expression was assessed by Westernblotting in A549 lung epithelial cells from Normal control group, LPS intervention group, PFOS intervention group, Nrf2 Inhibitor ML385 intervention group.

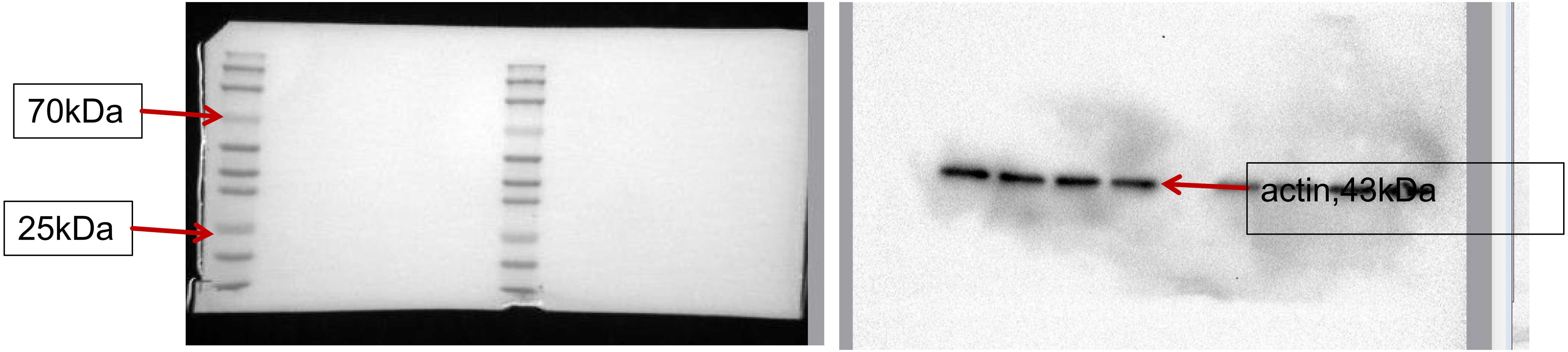

Figure 5C The molecular weight of actin is 43kDa.

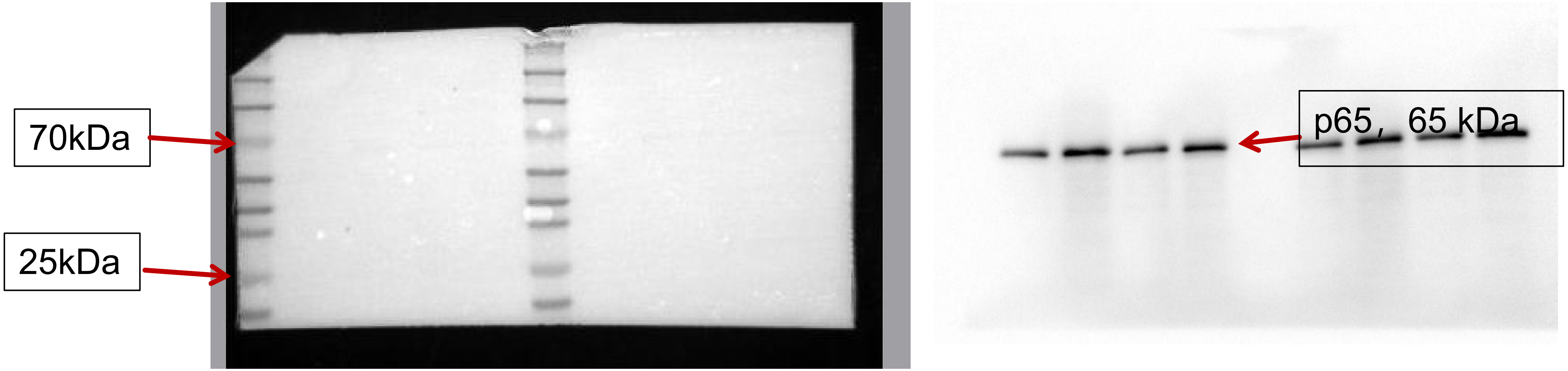

Figure 5E P65 is the key component of NFkB signal path, p65 expression was assessed by Westernblotting in A549 lung epithelial cells from Normal control group, LPS intervention group, PFOS intervention group, Nrf2 Inhibitor ML385 intervention group.

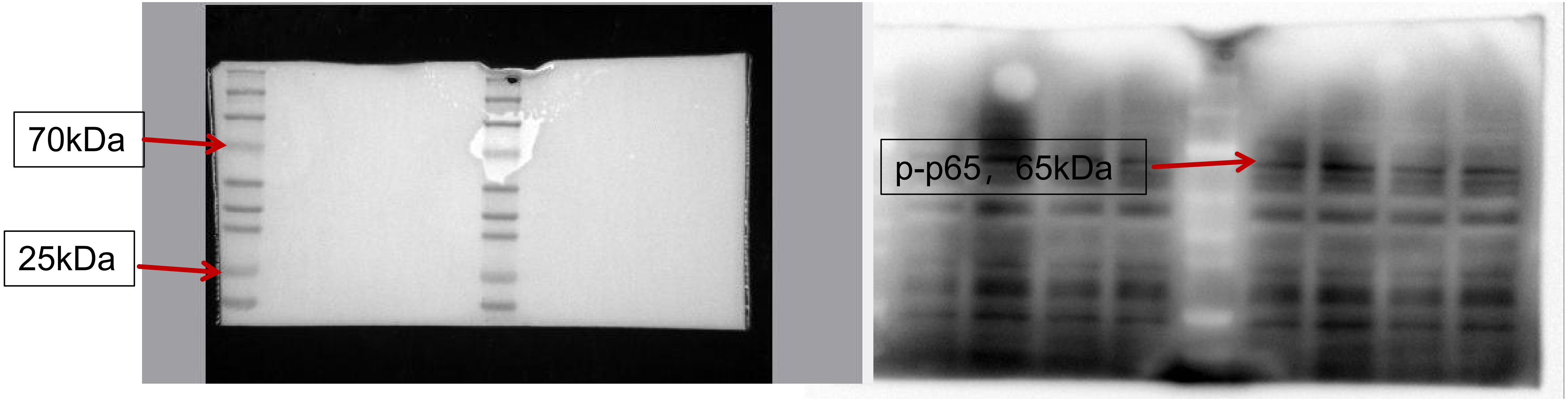

Figure 5E p-p65 is the key component of NFkB signal path, p-p65 expression was assessed by Westernblotting in A549 lung epithelial cells from Normal control group, LPS intervention group, PFOS intervention group, Nrf2 Inhibitor ML385 intervention group.

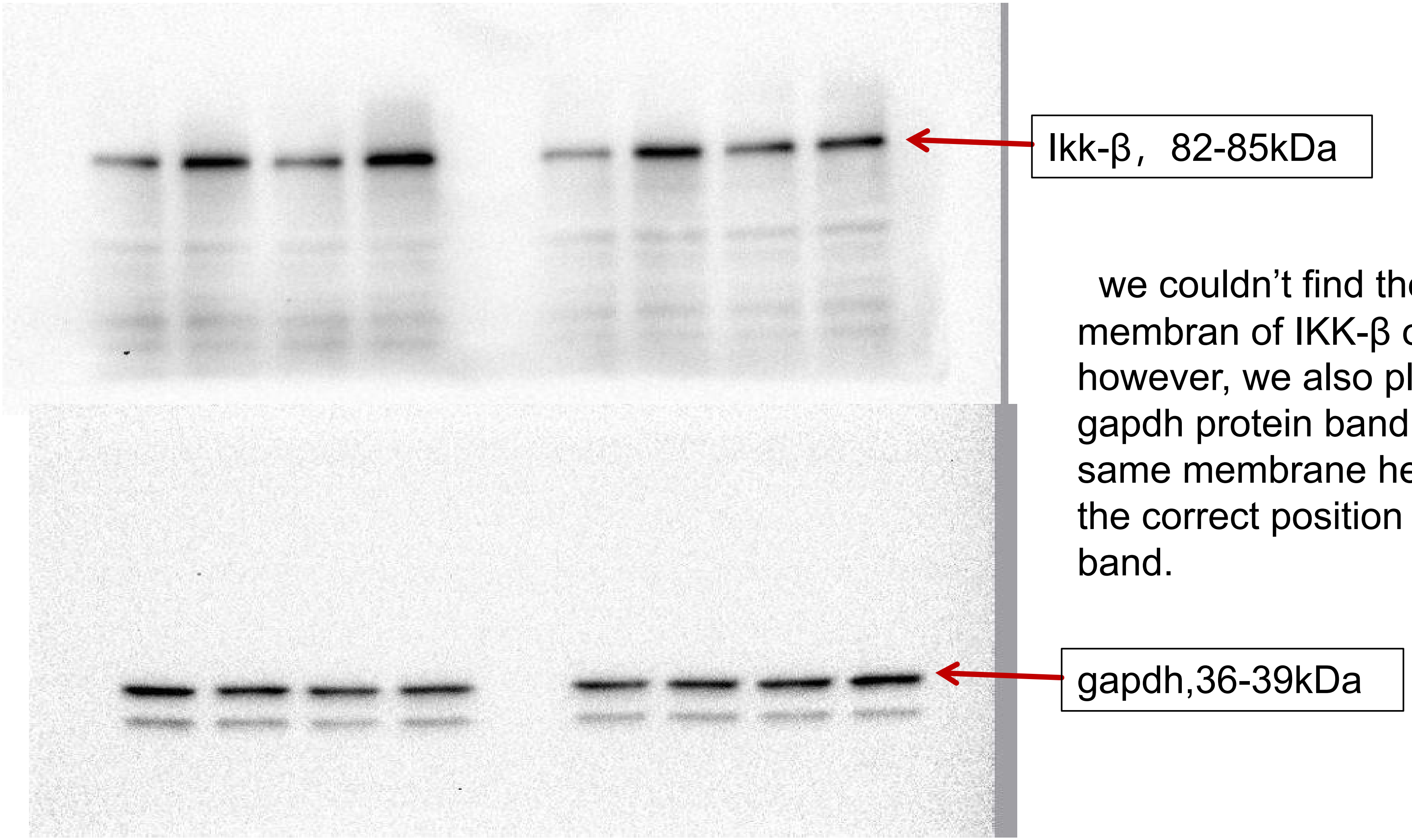

we couldn’t find the white membran of IKK-β of Figure5E， however, we also placed the gapdh protein band from the same membrane here to verify the correct position of the actin band.

Figure 5E Ikk-β is an other key component of NFkB signal path, Ikk-β expression was assessed by Westernblotting in A549 lung epithelial cells from Normal control group, LPS intervention group, PFOS intervention group, Nrf2 Inhibitor ML385 intervention group.

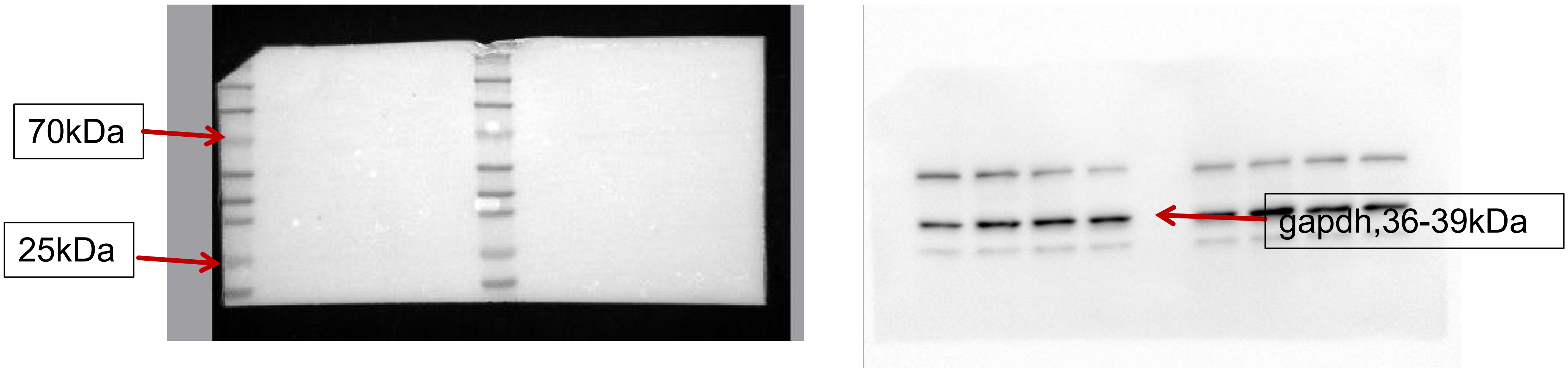

Figure 5E The molecular weight of Gapdh is 36-39 kDa.

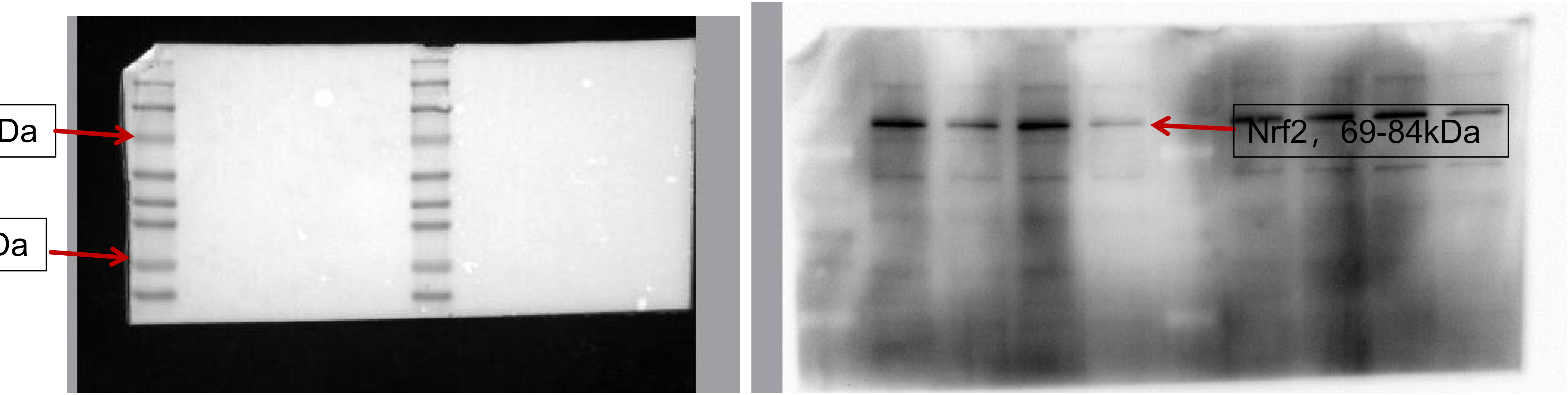

Figure 5G Nrf2 is a key protein in the oxidative stress signaling pathway, Nrf2 expression was assessed by Westernblotting in A549 lung epithelial cells from Normal control group, LPS intervention group, PFOS intervention group, Nrf2 Inhibitor ML385 intervention group.

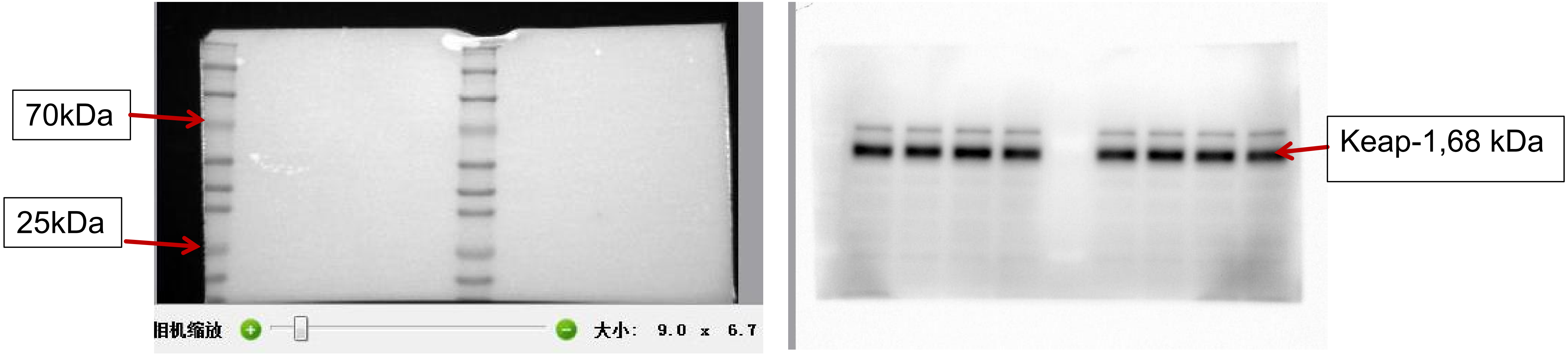

Figure 5G Keap-1 is a key protein in the oxidative stress signaling pathway, Keap-1 expression was assessed by Westernblotting in A549 lung epithelial cells from Normal control group, LPS intervention group, PFOS intervention group, Nrf2 Inhibitor ML385 intervention group.

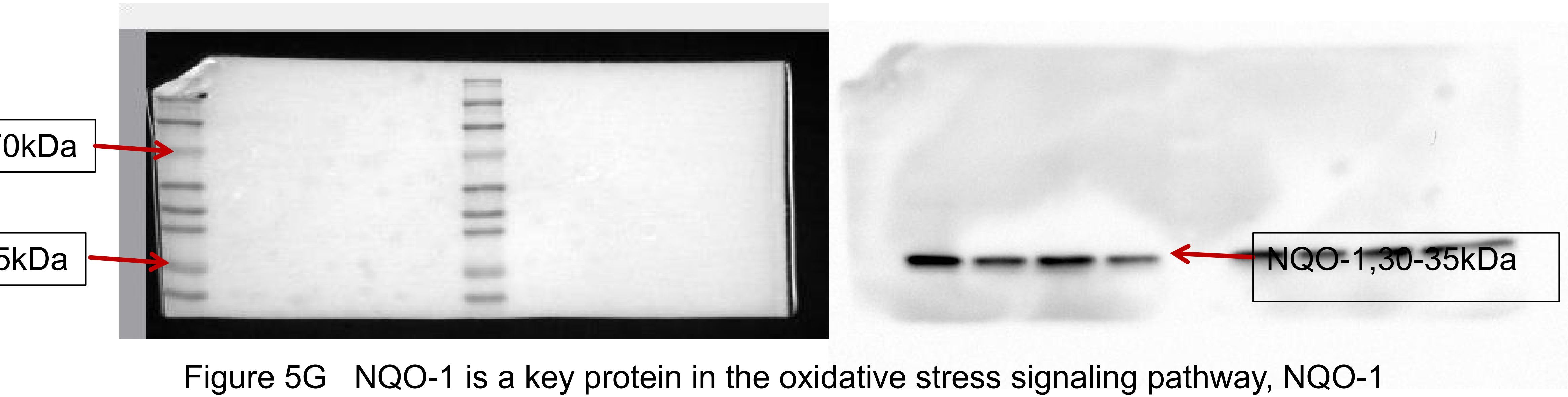

Figure 5G NQO-1 is a key protein in the oxidative stress signaling pathway, NQO-1 expression was assessed by Westernblotting in A549 lung epithelial cells from Normal control group, LPS intervention group, PFOS intervention group, Nrf2 Inhibitor ML385 intervention group.

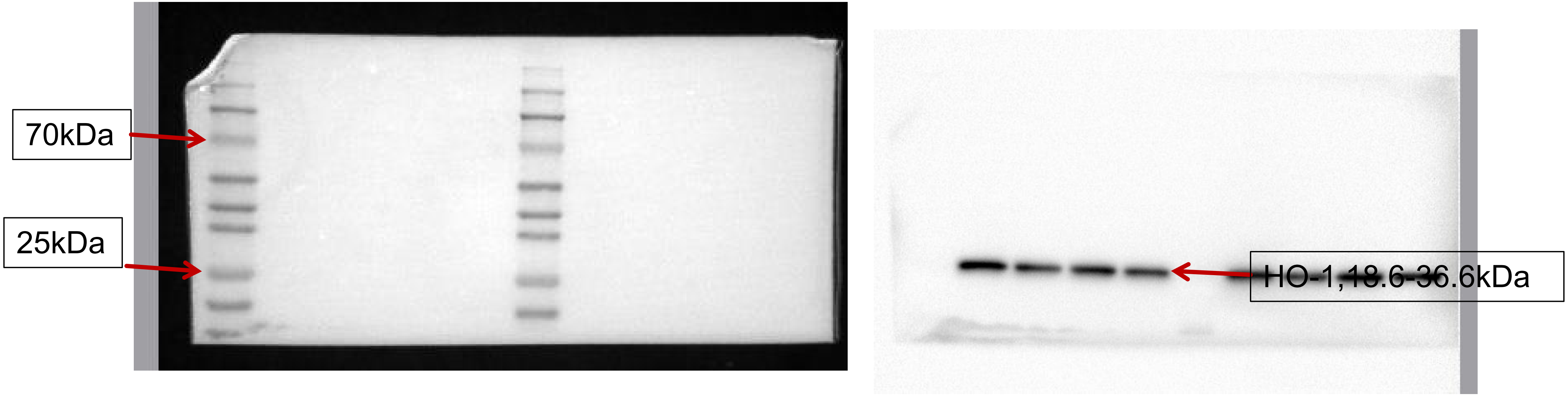

Figure 5G HO-1 is a key protein in the oxidative stress signaling pathway, HO-1 expression was assessed by Westernblotting in A549 lung epithelial cells from Normal control group, LPS intervention group, PFOS intervention group, Nrf2 Inhibitor ML385 intervention group.

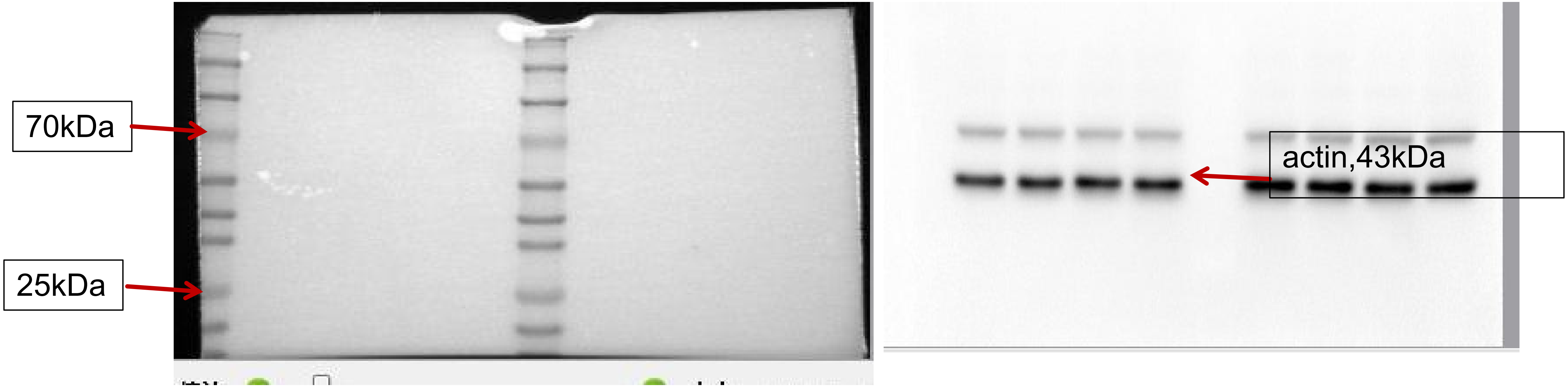

Figure 5G The molecular weight of actin is 43kDa.
