# Supplementary material for: Oligosaccharides from Polygonatum cyrtonema Hua ameliorate colitis-induced lung injury via modulation of the gut-lung axis through NF-κB and Nrf2 pathways
Source: Nat Prod Bioprospect. 2026 Jun 23;16(1):70. doi: 10.1007/s13659-026-00608-0 (PMC13291371; doi:10.1007/s13659-026-00608-0)
Supplement: Supplementary file 2 — Supplementary material 2. [file 13659_2026_608_MOESM2_ESM.docx]

**Supplementary Materials**

Supplementary Table S1

| *IL-1β-F* | 5’-ACTACAGGCTCCGAGATGAACAAC-3’ |
| --- | --- |
| *R* | 5’-CCCAAGGCCACAGGTATTTT-3’ |
| *IL-6-F* | 5-’CACATGTTCTCTGGGAAATCG-3’ |
| *R* | 5’-TTGTATCTCTGGAAGTTTCAGATTGTT-3‘ |
| *TNF-α-F* | 5’-ACGGCATGGATCTCAAAGAC-3’ |
| *R* | 5’-AGATAGCAAATCGGCTGACG-3’ |
| *GADPH-F* | 5’-GTGTTCCTACCCCCAATGTGT-3’ |
| *R* | 5’- ATTGTCATACCAGGAAATGAGCTT-3’ |
| *SOD 1- F* | 5’-TAACTGAAGGCCAGCATGGGT-3’ |
| *R* | 5’-GGTCTCCAACATGCCTCTCTTC-3’ |
| *GPX2 -F* | 5’-GAACGAGGAGATCCTGAACAGC-3’ |
| *R* | 5’-GGTAGGGCAGCTTGTCTTTCAG-3’ |
| *CAT- F* | 5’-TTGTTCAGTGACCGAGGGATT-3’ |
| *R* | 5’-TTCCTGAGCAAGCCTTCCTG-3’ |

Supplementary Table S2A

**
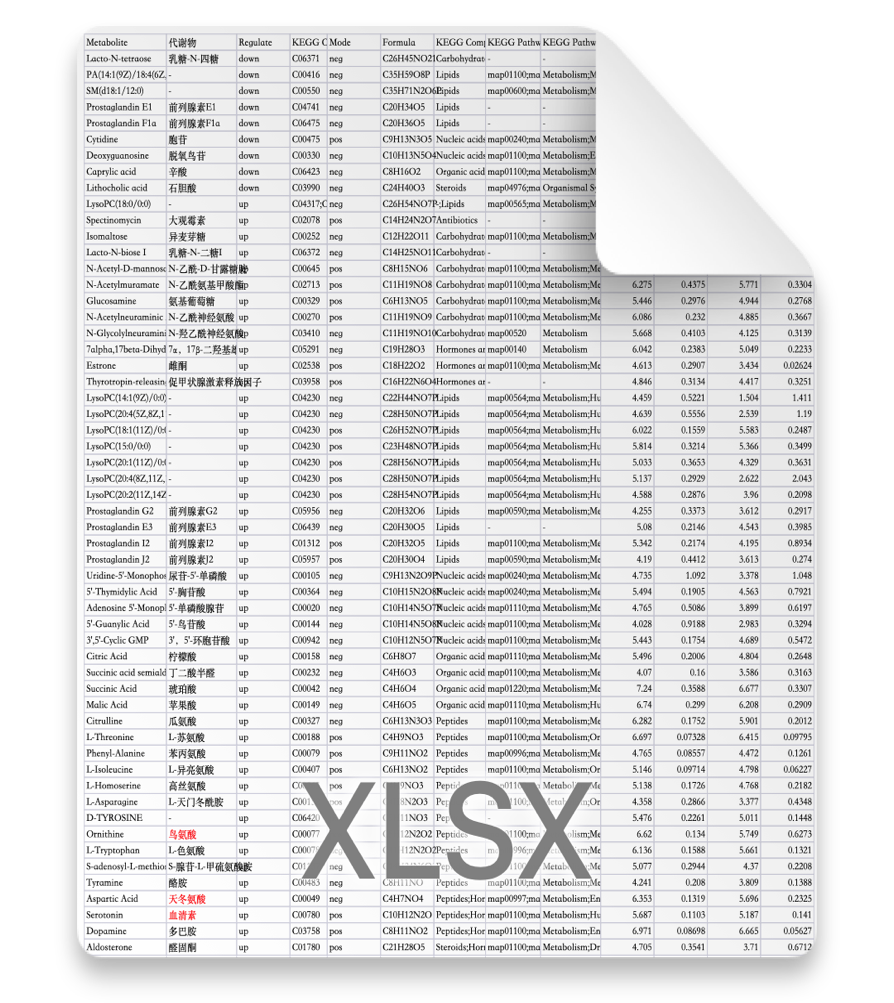
**Supplementary Table S2A shows that the significantly different metabolites in the normal control group and the DSS model group were annotated by KEGG and classified at the KEGG compound level, totaling 60 differentially expressed metabolites (the screening condition was VIP ≥ 1, P < 0.05). These differentially expressed metabolites mainly include 15 lipid compounds, including 6 twenty-alkane compounds (prostaglandin E1, prostaglandin F1a, prostaglandin G2, etc.), 9 different carbon-chain lysophosphatidylcholines ((LysoPC(18:1(11Z)/0:0), LysoPC(15:0/0:0), LysoPC(20:1(11Z)/0:0), etc.); 8 carbohydrates including oligosaccharides (lactose-N-tetraose, lactose-N-diglycoside,etc.) and monosaccharides (N-acetyl-D-mannosamine, N-acetylaminomethylate, glycoprotein, etc.); 7 nucleotide compounds (5'-thymidine acid, 5'-monophosphate adenosine, 5'-guanosine acid, etc.); 5 organic acid compounds (succinic semialdehyde, succinic acid, malic acid, etc.); 14 amino acids and their derivatives (L-threonine, ornithine, L-tryptophan, S-adenosyl-L-methionine amine, etc.); 3 vitamins and cofactors (folic acid, flavin mononucleotide, nicotinamide adenine dinucleotide); 6 steroidal substances (7α, 17β-dihydroxyandrosta-4-ene-3-one, estrone, thyroid stimulating hormone-releasing factor). Among them, 9 differentially expressed substances are downregulated, and 51 differentially expressed substances are upregulated. Almost all amino acids, carbonic compounds, and organic acid compounds show upregulation; the downregulated differentially expressed substances

mainly include 2 phospholipids, 2 twenty-alkane compounds, and 2 nucleotides.

Supplementary Table S2B

**
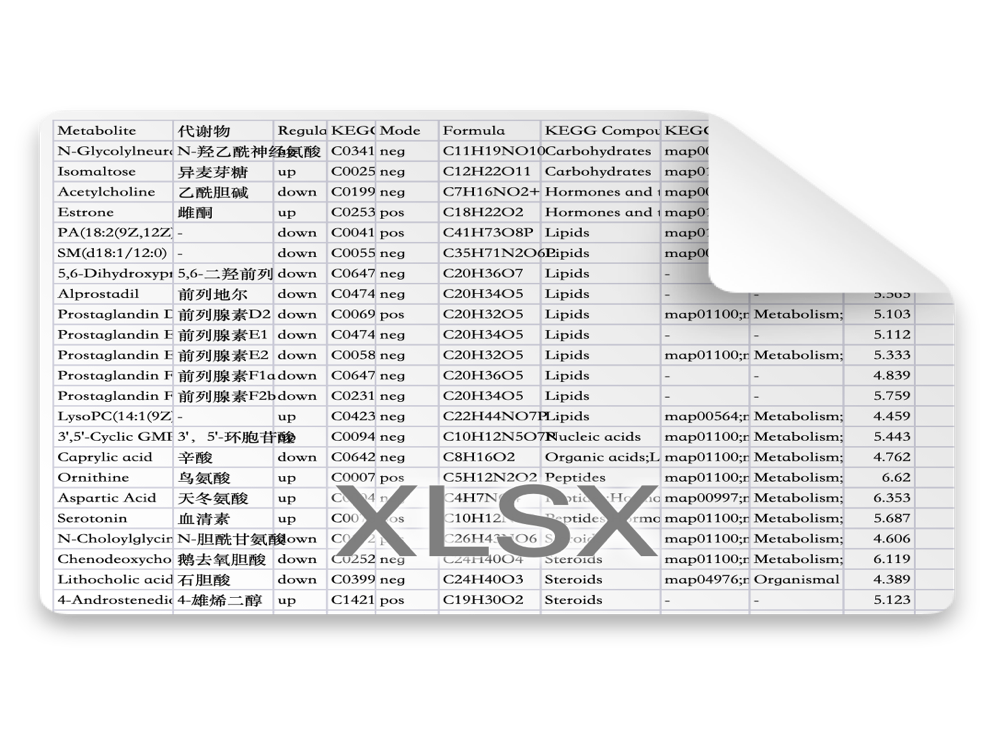
**

Supplementary Table S2B shows that 24 different metabolites with significant differences between the PFOS pre-intervention experimental group and the DSS model group were annotated by KEGG, and they were classified at the KEGG compound level (the screening conditions were VIP ≥ 1 and P < 0.05). Among them, there were 2 types of carbohydrates (isomaltose, N-hydroxyethylamine, etc.), 9 ester substances (prostaglandin F2b, prostaglandin E1, prostaglandin F1a, etc.), 3 amino acids and their derivatives (ornithine, serotonin, aspartic acid, etc.), 4 steroidal compounds (stearic acid, 4-cholesten-3-one, N-acylglycine, etc.), 1 nucleotide (3',5'-cyclic cytidine acid), 1 organic acid (octanoic acid), and 1 vitamin (pyridoxol).

Supplementary Fig.1

| 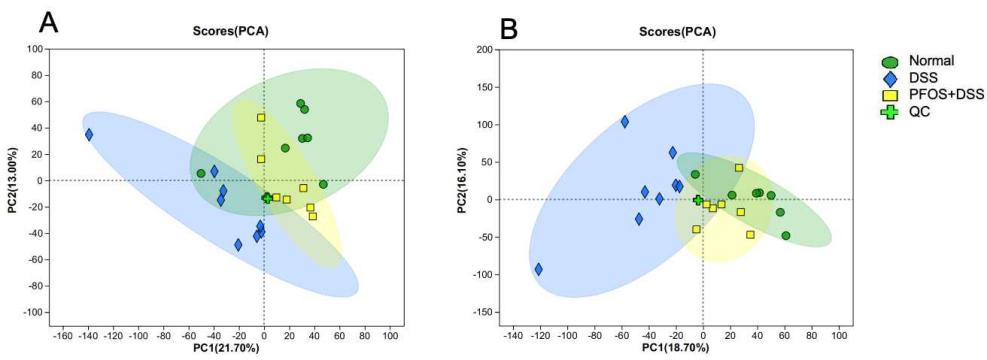 |
| --- |

Supplementary Fig.1 Raw data pre-processing and principal component analysis. (A, B) Cationic and anionic PCA analyses of fecal samples from the normal control group (a), DSS model group (b), and flavispermine oligosaccharide pre-intervention group.

Supplementary Fig.2

| 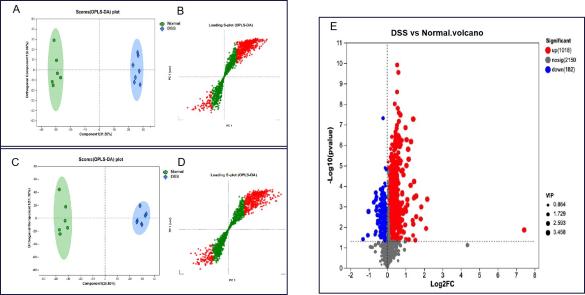 |
| --- |

Supplementary Fig.2 Analysis of different metabolites between DSS and normal control groups. A and B represent cationic and anionic OPLS-DA scores of normal control group (a) compared with DSS model group (b); C and D represent S-plots in positive and negative ion modes generated by differential metabolites according to p<0.05, VIP>1.0.

Supplementary Fig.3

| 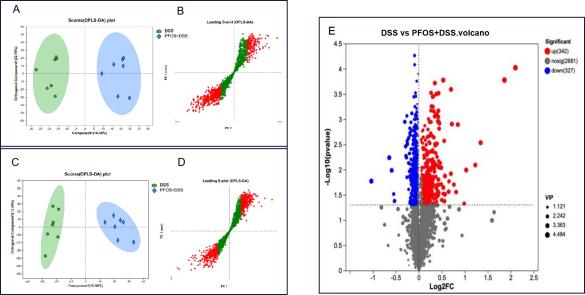 |
| --- |

Supplementary Fig.3 Multivariate statistical analysis of fecal metabolites in the PFOS pre-intervention and DSS model groups. A and B represent the cationic and anionic OPLS-DA scores of the PFOS pre-intervention group (c) and the DSS model group (b). C and D represent S-plots in positive and negative ion modes generated by differential metabolites according to p<0.05, VIP>1.0.
